# Neoplasms and tumor-like lesions of the sellar region: imaging findings with correlation to pathology and 2021 WHO classification

**DOI:** 10.1007/s00234-023-03120-1

**Published:** 2023-02-17

**Authors:** Lorenzo Ugga, Raduan Ahmed Franca, Alessandra Scaravilli, Domenico Solari, Sirio Cocozza, Fabio Tortora, Luigi Maria Cavallo, Marialaura Del Basso De Caro, Andrea Elefante

**Affiliations:** 1 Department of Advanced Biomedical Sciences, University of Naples “Federico II”, Naples, Italy; 2 Department of Neurosciences, Reproductive and Odontostomatological Sciences, University of Naples “Federico II”, Naples, Italy

**Keywords:** Pituitary neoplasms, Diagnostic imaging, Pathology

## Abstract

The sellar region represents a complex anatomical area, composed of multiple structures of different embryological derivation, including the skull base and the pituitary gland, along with vascular, nervous, and meningeal structures. Masses arising in this region include benign and malignant lesions arising from the pituitary gland itself, but also from vestigial embryological residues or surrounding tissues, that may require different therapeutic approaches. While assessing sellar region masses, the combination of clinical presentation and imaging features is fundamental to define hypotheses about their nature. MR represents the imaging modality of choice, providing information about the site of the lesion, its imaging features, and relation with adjacent structures, while CT is useful to confirm the presence of lesion calcifications or to reveal tumor invasion of bony structures. The aim of this pictorial review is to provide an overview of the common neoplasms and tumor-like conditions of the sellar region, according to the 2021 WHO Classification of Tumors of the Central Nervous System (fifth edition), with an emphasis on the radiologic-pathologic correlation. After a brief introduction on the anatomy of this region and the imaging and pathological techniques currently used, the most relevant MRI characteristics, clinical findings, and pathological data, including histologic and molecular features, will be shown and discussed, with the aim of facilitating an appropriate differential diagnosis among these entities.

## Introduction

The sellar region represents a complex anatomical area that includes the bony framework of the central skull base, the pituitary gland, and a series of vascular, nervous, cerebrospinal fluid (CSF), and meningeal structures surrounding it. Therefore, a neoplastic or tumor-like mass in this region can originate from any of these elements, and this explains the wide variety of differential diagnoses that can be postulated.

The classification of sellar region tumors is constantly evolving, with the current reference (namely, the 2021 WHO Classification of Tumors of the Central Nervous System–fifth edition), that introduces some changes to previous editions [1, 2], such as the classification of adamantinomatous and papillary craniopharyngiomas as different entities, or the inclusion of pituicytoma, granular cell tumor, and spindle cell oncocytoma in the same group of neoplasms. Additional changes are also the introduction of the new term pituitary neuro-endocrine tumor (PitNET) to define pituitary adenomas (PAs), which are now classified according to their adenohypophyseal cell-lineage, or the description of a new entity, the pituitary blastoma, which represents a rare embryonal neoplasm of infancy. Despite the broad spectrum of possible neoplastic and tumor-like entities, in almost 80% of cases lesions arising from this region are macroadenomas, aneurysms, craniopharyngiomas, meningiomas, or astrocytomas, with other lesions being relatively rare [3]. Diagnostic workup and subsequent management of these conditions often requires a multidisciplinary approach with a team including but not limited to neuroradiologists, neurosurgeons, endocrinologists, oncologists, and pathologists. From a neuroradiological standpoint, the modality of choice for the study of the sellar region is obviously magnetic resonance imaging (MRI), which is able to provide accurate and reliable information about the nature of the lesion, as well as its relationship with the adjacent structures, allowing to formulate proper hypotheses about its nature. Nevertheless, computed tomography (CT) is useful in some cases to confirm the presence of lesion calcifications, if any, or to better evaluate the lesion invasion of the adjacent bone [4].

Given the relative anatomical complexity of this region, and the recent release of the latest classification of CNS tumors, the aim of this review is to provide a review of the anatomy, imaging techniques, and most relevant MRI characteristics of neoplasms and tumor-like lesions involving the sellar region, together with clinical findings and pathological data, to help neuroradiologists in achieving an appropriate differential diagnosis among these entities.

### Anatomy

**Fig. 1:**
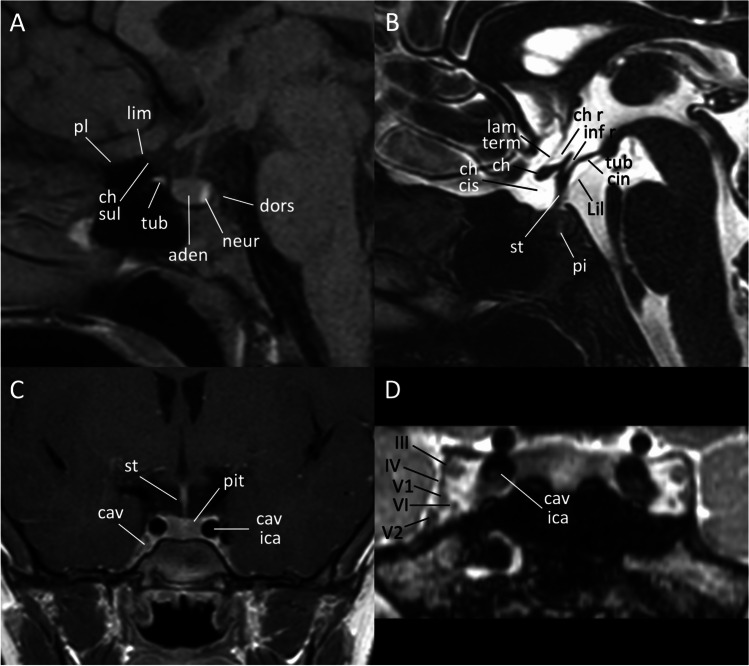
MR anatomy of the sellar region. Sagittal T1- (**A**), 3D high resolution T2- (sagittal reformat, **B**) and contrast-enhanced coronal T1-weighted (**C**) images; coronal reformat of a contrast-enhanced 3D balanced gradient echo sequence (**D**). *aden* adenohypophysis, *cav* cavernous sinus, *cav ica* cavernous segment of the internal carotid artery, *ch* optic chiasm, *ch cis* chiasmatic cistern, *ch r* chiasmatic recess, *ch sul* chiasmatic sulcus, *dors* dorsum sellae, *inf r* infundibular recess, *lam term* lamina terminalis, *Lil* Liliequist membrane, *lim* limbus sphenoidale, *neur* neurohypophysis, *pi* pars intermedia, *pit* pituitary gland, *pl* planum sphenoidale, *st* pituitary stalk, *tub* tuberculum sellae, *tub cin* tuber cinereum, *III* third cranial nerve, *IV* fourth cranial nerve, *V1* ophthalmic branch of the fifth cranial nerve, *V2* maxillary branch of the fifth cranial nerve, *VI* sixth cranial nerve

The sella turcica is a saddle-shaped depression on the basisphenoid. The borders of this anatomical structure are defined anteriorly by the middle clinoid processes (two small eminences one on either side), while the posterior border is formed by the dorsum sellae which ends at its superior angles with the posterior clinoid processes (Fig. 1A). The sellar floor is part of the sphenoid sinus roof, which is covered by dura and separates it from the pituitary gland. The bony structures of the sella turcica are indeed overlayed by a dural reflection named the diaphragma sellae, which forms a roof over the sella that almost covers the pituitary gland. The diaphragma sellae has a variably sized central opening, the so-called diaphragmatic hiatus, that allows the passage of the pituitary stalk [5].

The sella turcica harbor the pituitary gland, a bean-shaped gland with two distinct lobes: the anterior lobe, also called the adenohypophysis, and the posterior one named neurohypophysis (Fig. 1A). The anterior lobe, by far the largest part of the gland, is in turn divided into a “pars tuberalis” (rich in capillaries), a “pars intermedia” and a “pars distalis” (being in terms of volume the most prevalent segment of this portion of the gland). From an embryological perspective, the pituitary gland has a dual origin which reflects the two distinct parts in the gland, with the Rathke’s pouch, a dorsal evagination of the stomodeum, that forms the anterior and intermediate lobes, while the infundibulum, a ventral extension of the diencephalon, forms the posterior lobe [6]. The anterior lobe is responsible for synthesis and release of most pituitary hormones, such as growth hormone (GH), corticotropin (ACTH), follicle-stimulating (FSH) and luteinizing hormones (LH), prolactin (PRL) and thyrotropic hormone (TSH), with the only exception of oxytocin and antidiuretic hormone (ADH) which are released by the posterior lobe. The pituitary gland is anatomically and functionally related to the hypothalamus by a stem called the infundibulum, or pituitary stalk. The stalk extends inferiorly from the tuber cinereum, which is a part of hypothalamus and a thin convex mass of gray matter that lies between the optic chiasm anteriorly and the mammillary bodies posteriorly and descends to become continuous with the posterior pituitary lobe (Fig. 1B, C); the third ventricle lies in the midline just above the hypothalamus. Along the boundaries of the third ventricle, there are four small outpocketings called recesses, two of them are located anteriorly adjacent to the sellar region [7]. These are the supraoptic recesses, above the optic chiasm, and the infundibular recess in the pituitary stalk. The supraoptic recess is more rounded while the infundibular recess is more conical and pointed (Fig. 1B). A thin dural reflection borders the pituitary fossa laterally and forms the medial cavernous sinus wall. Cavernous sinus contains the cavernous segment of internal carotid artery (ICA) and several cranial nerves that pass through the lateral dural wall. Of these, the most cranial one is the oculomotor nerve (III), with the trochlear (IV) that lies just below it. Two division of the trigeminal nerve, the ophthalmic (V1) and maxillary (V2) divisions, are located inferiorly to the IV nerve, while the abducens (VI) is the only cranial nerve that lies within the cavernous sinus, inferolateral to the cavernous tract of the ICA (Fig. 1D).

### Imaging technique

The gold-standard imaging modality for sellar region assessment is undoubtedly MRI, that should be performed on high-field scanners (1.5 T or higher) with a multi-channel head coil allowing for parallel imaging. Standard MR protocol should include turbo spin echo (TSE) T1- and T2-weighted sequences acquired on both the sagittal and coronal planes, using a small (13–18 cm) field of view (FOV) with a slice thickness less than or equal to 3 mm, with axial images that might provide additional information on lesion morphology and extension in selected cases.

Gadolinium-based contrast agent administration is always recommended in the characterization of neoplasm or tumor-like lesions of the sellar region, followed by TSE T1-weighted sequences similar to those acquired prior to the contrast administration; furthermore, in a few conditions, it may not be necessary, such as the growth and puberty disorders (GPD) in the pediatric population [8]. Dynamic contrast-enhanced (DCE) coronal images during contrast injection with a temporal resolution of at least 20 s are mandatory whether a clinical/laboratory suspicion of a pituitary microadenoma is present [4, 9], although its acquisition might be also useful in other scenarios, such as in the follow-up of treated adenomatous lesions [10].

Optional sequences that can be acquired for the study of the sellar region include diffusion-weighted imaging (DWI), susceptibility-weighted imaging (SWI), volumetric high-resolution T1- and T2-weighted sequences, and MR angiography (MRA). In particular, DWI provide information about lesion cellularity, by measuring water molecule diffusion movements, and recent studies have shown that apparent diffusion coefficient (ADC) values correlate with tumor consistency and proliferative index in PAs, even if these data require further validation [11, 12]. Nevertheless, it should be stressed that clinical routinely acquired DWI is usually based on echo-planar imaging technique, and that the sequence is therefore severely affected by the air-tissue interface artifacts commonly present adjacent to skull base [13]. However, non-EPI DWI, such as TSE-DWI, are less sensitive to susceptibility artifacts and represent therefore a possible better sequence for sellar region assessment [14]. On the other hand, SWI is useful in depicting intralesional calcification or hemorrhage, being able to distinguish these two entities based on the evaluation of both magnitude and phase maps [15]. Nevertheless, also SWI is limited by the above-mentioned air-tissue interface artifacts. The acquisition of contrast-enhanced volumetric T1-weighted sequences with fat saturation (i.e., VIBE, THRIVE and LAVA sequences) is strongly recommended if a skull base involvement is found on pre-contrast images, while three-dimensional high-resolution T2-weighted sequences better delineate the pituitary stalk, suprasellar cistern and cystic lesions. In addition, balanced gradient-echo sequences (i.e., CISS, FIESTA and b-TFE sequences), in which image contrast varies according to the T2/T1 ratio, can be collected after contrast agent administration to take advantage of both fluid and gadolinium contrasts [16], with this approach being useful for cavernous sinus and related cranial nerves investigation if obtained on the coronal plane.

Perfusion-weighted imaging (PWI) can provide additional information helpful in differential diagnosis of sellar/parasellar tumors. Indeed, although both PAs and meningiomas are lesions with a significant vascularization, meningiomas show significantly higher relative cerebral blood volume values compared to PAs [17]. Furthermore, PWI also allows differentiating these latter lesions from other sellar tumors, such as craniopharyngioma or hemangioblastoma, which in some cases might be mimickers on conventional MRI scans [17]. Finally, MRA can be performed with or without contrast administration and defines size and course of intracranial arteries, which may be displaced and compressed by a sellar lesion.

Although MRI represents the imaging gold-standard for the diagnosis of sellar lesions, CT might provide supportive information regarding the anatomy of the sphenoid bone and sinus, also revealing and depicting the degree of tumor invasion of adjacent bony structures. Furthermore, the acquisition of CT images might confirm the presence of lesion calcifications suspected using SWI sequences. Finally, CT angiography might help in better describing the relationships between intracranial arteries and the lesion, and a CT of the brain may still be helpful in the emergency room to exclude the presence of an acute pituitary hemorrhage.

### Pathological examination basics

The first and probably most important question a pathologist should answer is whether the observed sample contains tissue from a normal pituitary gland or not. Although in typical cases PA morphology is straightforward, and is therefore relatively easy to achieve a correct diagnosis, unusual morphological patterns might be present for other lesions (including but not limited to sinusoidal pattern, macronodular or festoon-like features, as well as lesions with diffuse epithelioid features) and might therefore cause diagnostic concerns [18]⁠. In these cases, the routinely immunohistochemical panel performed (i.e., ACTH, PRL, GH, TSH, FSH, LH, GH, Ki67), as well as reticulin staining, markers of neuroendocrine differentiation (e.g. chromogranin or synaptophysin) and immunoreactivity for transcription factors (e.g., T-Pit, Pit1 or SF1) are all useful markers commonly employed to achieve a correct diagnosis [19]⁠. Among these, the most helpful tool to differentiate adenomas from normal adenohypophysis parenchyma, as well as from hyperplastic lesions, is reticulin staining [20], given that the normal anterior pituitary gland retains reticulin pattern, which is expanded in pituitary hyperplasia and disrupted in adenomas. On the other hand, the differential diagnosis between a PA and a metastatic cancer is only rarely a concern, given that mitotic activity and cellular pleomorphism are nearly always hallmarks of a metastatic neoplasm while being extremely rare in PAs. Nevertheless, when a lesion is thought to be a metastasis, additional markers should be performed to best define the primary tumor lineage. In particular, LCA (CD45) positivity is usually observed in lymphoproliferative lesions, while carcinomas are in almost all cases pancytocheratin positive. The positivity to TTF1 suggests a pulmonary or thyroid origin, with the latter being also positive for thyroglobulin and PAX8, while CDX2 positivity might suggest a cancer originating in gastroenteric tract. Other examples are the positivity to HMB45, MART1, SOX10, or S100 immunoreactivity in cases of melanoma, GCDFP-15 and mammaglobin for breast cancer lesions, or PAX8 immunoreactivity for lesions originating from renal cell carcinoma [21].

A particular setting is the evaluation of a lesion immunonegative for all the pituitary markers such as hormones and transcription factors, but positive for neuroendocrine markers, raising the possibility of a differential diagnosis between a null cell adenoma and metastatic neuroendocrine carcinoma. In those cases, lesion morphology, mitotic activity, and lineage differentiation markers (e.g., TTF1, CDX2, CK, calcitonin, etc.) might help in making a correct diagnosis. However, it must be kept in mind the possibility of the presence of relatively rare and highly debated entities such as the primary intracranial neuroendocrine carcinoma arising in sellar region (which is TTF1−)⁠ or the small cell carcinoma of unknown primary (SCUP, which is TTF1+) [22]⁠.

Finally, lesions purely cystic or with some cystic components (e.g., craniopharyngiomas or Rathke cleft cysts) rarely represent a diagnostic dilemma from a pathological standpoint, given the relation between their clear morphology and typical radiological features. In this light, it is noteworthy to stress that the cystic appearance is a phenomenon clearer to neuroradiologist rather than to pathologist.

### Pituitary adenoma

PAs, that following the 2021 WHO Classification of Tumors of the Central Nervous System are now known as PitNETs, represent the most common masses of the sellar region in adults arising from the adenohypophysis [23], with a peak of presentation between the 4th and 7th decade. About 5% of PAs are associated with familiar tumor syndromes, such as multiple endocrine neoplasia type 1, Carney complex, McCune-Albright syndrome, and familial isolated pituitary adenoma syndrome [24]. Rare ectopic sites of PAs include sphenoid sinus, nasopharynx, third ventricle, and suprasellar cistern [25]. PAs are classified based on their size as macroadenomas (≥ 10 mm) or microadenomas (< 10 mm), while giant adenoma is a term used to describe masses bigger than 40 mm [26]. PAs can also be classified as functioning or non-functioning (NFPAs), according to their capability of secreting hormones, with two-third of PAs that clinically present with a hypersecretory syndrome [27]. In particular, lactotroph adenomas in young female patients are generally microadenomas and manifest with amenorrhea and galactorrhea, while hypogonadism and impotence in male patients and delayed puberty in children tend to manifest for higher level of serum prolactin, related to macroadenomas [28]. Somatotroph adenomas are usually macroadenomas and present with acromegaly in adults and gigantism in children, while corticotroph adenomas are more frequently microadenoma (or picoadenomas, < 3–4 mm) [29] and often present as Cushing disease in women at 30–50 years, being also the most common type of PA in children [30]. Finally, thyrotroph adenomas, presenting with hyperthyroidism, are often macroadenomas with extrasellar extension [31]. On other hand, about one-third of PAs are NFPAs, and usually manifest as macroadenomas with suprasellar extension, either infra- or supradiaphragmatic, leading to symptoms that are almost exclusively related to their mass effect and include visual disturbance, headache, and hypopituitarism. Macroadenomas may rarely have an acute presentation with pituitary apoplexy, an endocrine emergency which can occur due to infarction or hemorrhage of the pituitary gland [32].

In the revised 2021 classification based on cell lineage, immunohistochemical analysis and transcription factors, PitNETs can also be classified arising from three cell lineages: TPIT (corticotroph), PIT1 (somatotrophs, lactotrophs, thyrotrophs), and SF1 (gonadotroph) lineage tumors, which can be clinical functioning or silent [33]. PIT1 is a complex group that includes tumors arising from a single cell population secreting one or multiple hormones, and tumors arising from different cell lines secreting two or more hormones. In addition to plurihormonal tumors of PIT1-lineage, there are also immature PIT1-lineage tumors. This group includes tumors expressing nuclear PIT1 without a terminal differentiation. They can be clinically silent or can secrete multiple hormones and tend to have aggressive behavior and poor prognosis [34]. Null cell adenomas present immunonegativity for pituitary transcription factors and hormones [35].

**Fig. 2:**
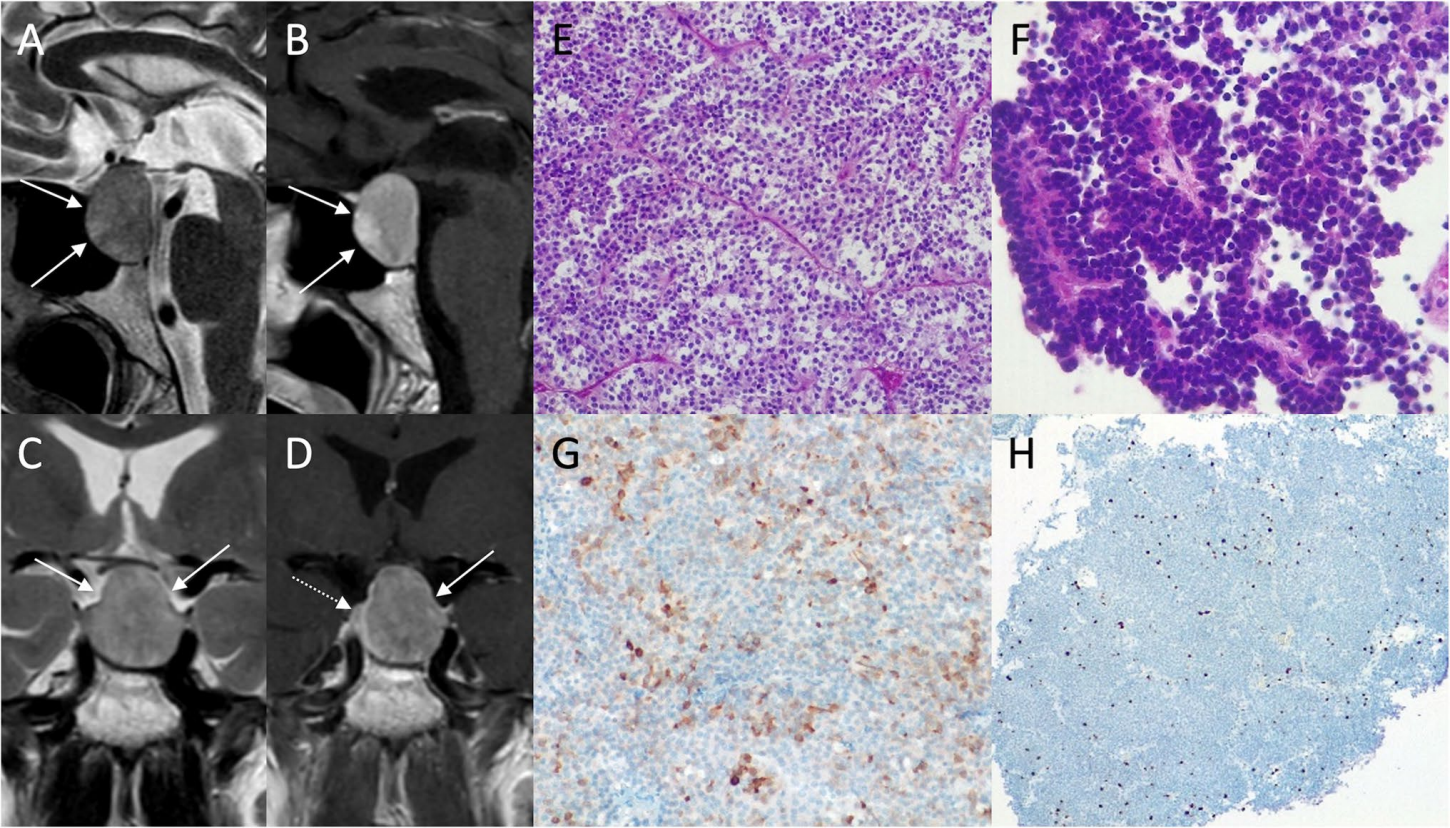
Pituitary macroadenoma. Sagittal T2- (**A**) and contrast-enhanced T1- (**B**), and coronal T2- (**C**), and contrast-enhanced T1-weighted (**D**) sequences show an intra- and suprasellar lesion leading to sellar enlargement and mild chiasmatic compression (arrows). It is not dissociable from the pituitary gland, which appears dislocated to the right (dotted arrow). Histological examination shows a tumor with solid growth pattern, that in some areas is composed of elongated cells arranged in a perivascular fashion (hematoxylin-eosin staining, original magnification 200×) (**E**). True papillae with fibrovascular core are also appreciable (hematoxylin-eosin staining, original magnification 400×) (**F**). Multifocal, scattered positivity for LH (immunoperoxidase staining, original magnification 200×) (**G**). Ki67 immunolabeling stains nearly to 2–3% of nuclei (immunoperoxidase staining, original magnification 100×) (**H**). These findings are consistent with pituitary adenoma/Pit-NET, LH-secreting

**Fig. 3:**
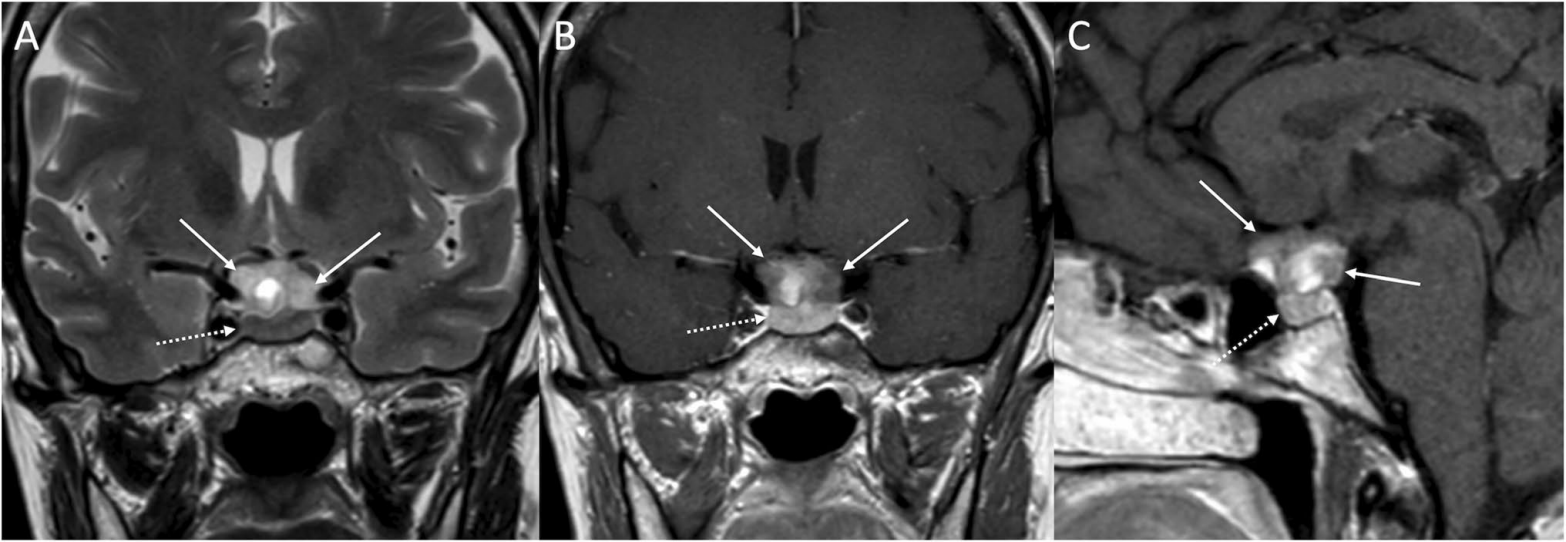
Suprasellar adenoma. Coronal T2- (**A**), contrast-enhanced coronal (**B**), and sagittal (**C**) T1-weighted images show a suprasellar lesion (arrows) dissociable from the underlying pituitary gland (dotted arrows)

On MRI, PAs generally present as sellar or intra- and suprasellar masses not clearly separated from the pituitary gland (Figs. 2 and 3). Generally, lactotroph and somatotroph adenomas arise from the lateral part of adenohypophysis, while thyrotroph and corticotroph adenomas arise in its central portion.

**Fig. 4:**
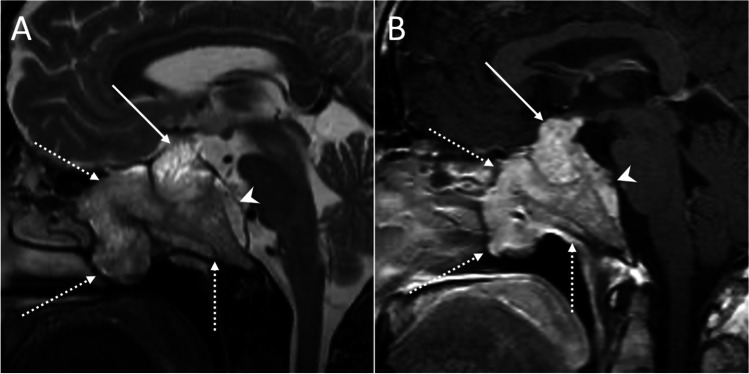
Invasive skull base pituitary adenoma. Sagittal T2- and contrast-enhanced T1-weighted images depict an intra- and suprasellar lesion (arrows) invading the adjacent central skull base (dotted arrows) and emerging to the retroclival epidural space (arrowheads)

**Fig. 5:**
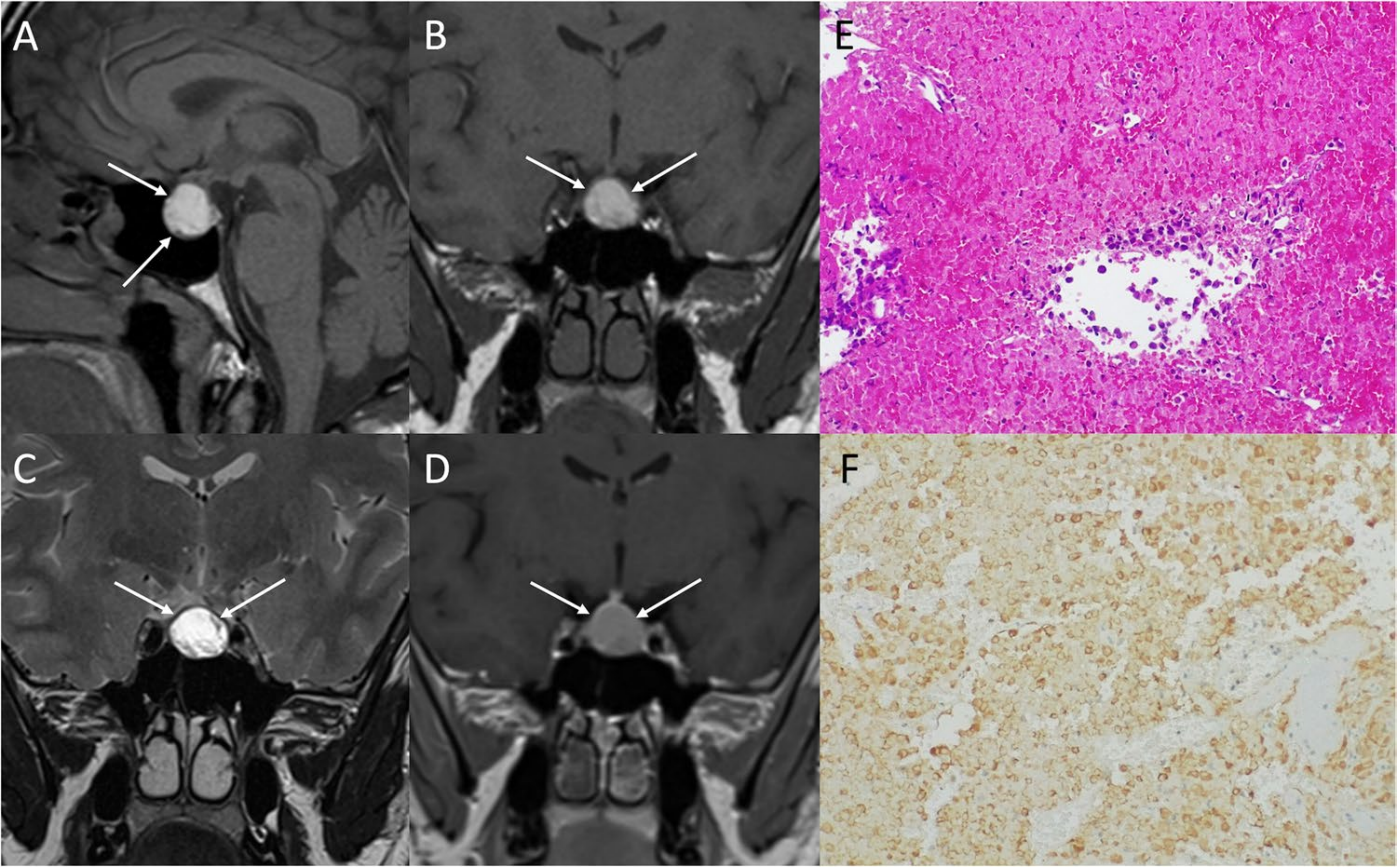
Pituitary apoplexy. Sagittal (**A**) and coronal (**B**) T1-, coronal T2- (**C**), and contrast-enhanced T1-weighted (**D**) sequences show an infradiaphragmatic inhomogeneous lesion featuring high T1-signal consistent with intralesional bleeding (arrows). Microscopic examination reveals a pituitary adenoma with widespread degenerative features (i.e., necrosis and hemorrhage) in patient with known acromegaly. Residual tumor cells are medium-sized, with hyperchromic and slowly pleomorphic nuclei and eosinophilic cytoplasm. The more “pink” areas are composed of “ghosts,” ischemic adenomatous cells, that are GH-immunoreactive on immunohistochemistry (**E**, hematoxylin-eosin staining, original magnification 200×; **F**, immunoperoxidase staining, original magnification 200×)

**Fig. 6:**
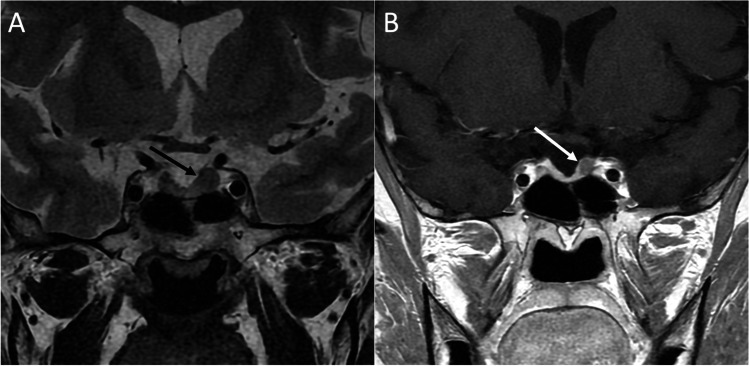
Growth hormone–secreting pituitary microadenoma. Coronal T2- (**A**) and contrast-enhanced T1-weighted (**B**) images show a mild thickening of the left aspect of the pituitary gland (black arrow) due to the presence of a small round lesion enhancing less than the remaining glandular parenchyma (white arrow)

Low T2-weighted signal intensity is typical of densely granulated somatotroph adenomas, that are more responsive to somatostatin analogs [36]. Ectopic pituitary adenomas are defined as PAs without any contact at all with the pituitary gland [37]. Invasive skull base PAs may also occur (Fig. 4). Pituitary apoplexy usually presents as a T1-hyperintense mass due to hemorrhage, with variable enhancement (Fig. 5). With reference to microadenomas, about 30% of these lesions are exclusively depictable using contrast-enhanced images, showing a delayed enhancement compared to the normal pituitary tissue appearing therefore slightly hypointense on post-contrast images (Fig. 6), while macroadenomas enhance strongly and heterogeneously and often present cysts, hemorrhagic foci, and sometimes a “dural tail.” Microadenomas are usually hypointense lesions on contrast-enhanced T1w images; however, one-third of them show high signal intensity on T2w images which makes them visible before contrast administration. Cavernous sinus invasion and suprasellar extension are signs of the invasiveness of macroadenomas, which is associated with worst prognosis [38]. The modified Knosp classification takes into account the relation between PA and intracavernous ICA, with a grading that scores from 0 (which indicates absence of cavernous sinus extension) to 4 (which indicates total encasement of intracavernous ICA), to assess surgical risk [39]. Bone remodeling of the skull base with sellar enlargement is often detected in larger adenomas using CT scans.

Histological examination reveals a tumor with solid growth pattern, in some areas composed of elongated cells arranged in a perivascular fashion. True papillae with fibrovascular core can also be appreciable.

Common differential diagnoses of PAs include pituitary hyperplasia, meningioma, metastasis, craniopharyngioma, and hypophysitis.

Furthermore, in some cases, the enlargement of the pituitary gland due to intracranial hypotension syndrome might be misinterpreted as a PA [40]. With reference to differential diagnosis with craniopharyngioma, it is noteworthy to mention that lactotroph adenomas are often prone to hemorrhage and the presence of a “fluid-fluid level” is highly suggestive of PAs rather than a craniopharyngioma [41, 42].

Advanced imaging techniques, such as MR elastography and textural analysis, have shown potential in predicting tumor consistency and treatment response before surgical resection [43]. The therapy of choice for PAs remains surgical resection, except for prolactinomas where the therapy is based on dopamine agonists, such as cabergolin and bromocriptine. Other treatment options include pharmacotherapy, stereotactic radiosurgery, and radiotherapy.

### Pituitary blastoma

Pituitary blastoma is a rare embryonal neoplasm arising from the fetal adenohypophysis, recently added as in the new 2021 WHO classification [1]. It usually present in children with less than 2 years with signs of Cushing disease, often related to a poor prognosis and part of DICER1 syndrome, a rare genetic disorder that predisposes patients to tumor development [44]. From an imaging standpoint, pituitary blastoma presents as an intra- and suprasellar mass with inhomogeneous enhancement, potentially invading the cavernous sinus and adjacent structures.

### Craniopharyngioma

Craniopharyngioma (CP) is a benign slow growing (WHO grade 1) sellar/suprasellar neoplasm that arises from the epithelial remnants in the anterior part of Rathke pouch at the infundibular stalk juncture with pituitary gland [45]. According to the 2021 WHO classification, CPs include two distinct entities, adamantinomatous CP (ACP) and papillary CP (PCP), formerly considered only subtypes, given that now is known that they exhibit different clinical and molecular features [46], and will be therefore discussed separately.

#### Adamantinomatous craniopharyngioma

ACP is significantly more common than PCP (90% vs. 10% of cases, respectively), and is usually more prevalent in children, although a bimodal age distribution has been observed with a first peak incidence between 5 and 15 years of age and the second ranging from 45 to 60 [47]. It frequently involves the third ventricle, optic chiasm, pituitary gland, and hypothalamus, with clinical manifestations related to the tumor size and mass effect that include headache, visual loss or hydrocephalus, as well as endocrine deficit (e.g., stunted growth, hypogonadism or central diabetes insipidus) [47].

**Fig. 7:**
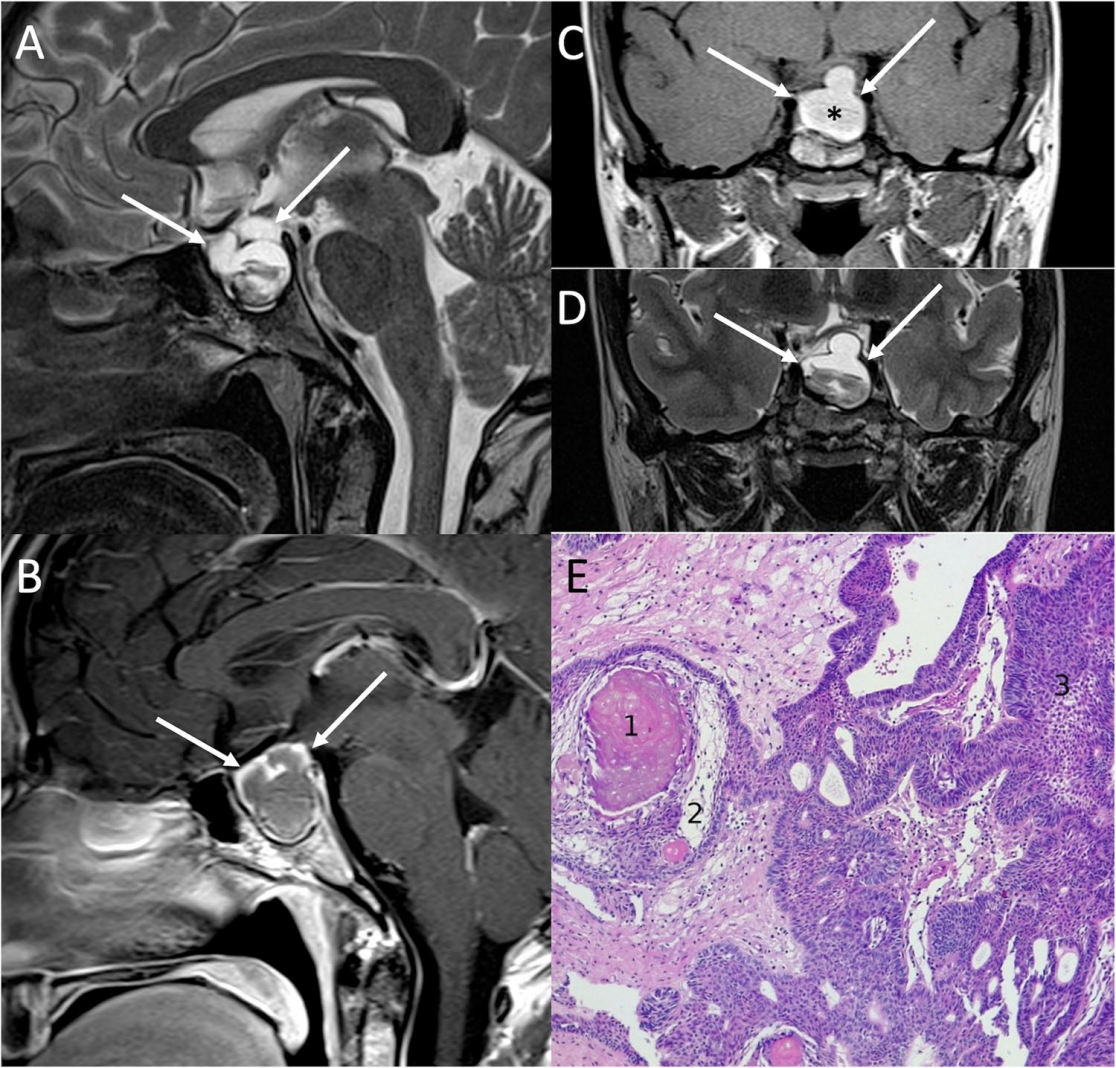
Adamantinomatous craniopharyngioma. Sagittal T2- (**A**) and contrast-enhanced T1- (**B**), and coronal T1- (**C**), and T2-weighted (**D**) MR images show an intra- and suprasellar multiloculated lesion (arrows) characterized by inhomogeneous content, mostly T1-hyperintense (asterisk), and peripheral enhancement. Microscopic examination (**E**) reveals keratin deposits, the so-called wet keratin (1), surrounded by loose textured cells, known as “stellate reticulum” (2). Reticulum is thought to derive from degeneration of internal cells. The cords and islands of well differentiated squamous epithelium surrounded by palisading columnar epithelium (3) are distinctive and diagnostic features of adamantinomatous craniopharyngioma (hematoxylin-eosin staining, original magnification 100×)

On imaging, ACP typically presents as a multilobulated mixed cystic-solid lesion, partially calcified, usually with supra- and/or infradiaphragmatic extensions and finger-like projections into neighboring structures [46]. Cystic components contain a dark, “motor oil-like” fluid, rich in cholesterol crystals [48], which reflects the appearance on MRI, with high-intensity signal on T1-weighted images that results from high protein content, presence of cholesterol crystals or methemoglobin. After gadolinium administration, enhancement of the cyst walls and solid components is typical (Fig. 7) [47]. Although SWI might provide information about calcifications, CT remains the gold standard to identify these features, which are thought to be due to osteoblastic differentiation of tumor cells [49]. Main differential diagnoses include cystic/hemorrhagic PAs, which however are rare in children, suprasellar epidermoid, dermoid, and arachnoid cysts that usually do not usually show peripheral contrast enhancement, and Rathke cleft cyst, which is usually a small, purely cystic, noncalcified lesion without nodular enhancement.

On histology, the hallmarks of ACP are keratin deposits (the so-called wet keratin), surrounded by loose textured cells known as “stellate reticulum,” thought to derive from degeneration of internal cells. The cords and islands of well-differentiated squamous epithelium surrounded by palisading columnar epithelium are distinctive and diagnostic features of ACP [50]. In some cases, ACP may evoke a florid gliotic reaction on the adjacent cerebral parenchyma, making differential diagnosis with a pilocytic astrocytoma more difficult [51].

#### Papillary craniopharyngioma

**Fig. 8:**
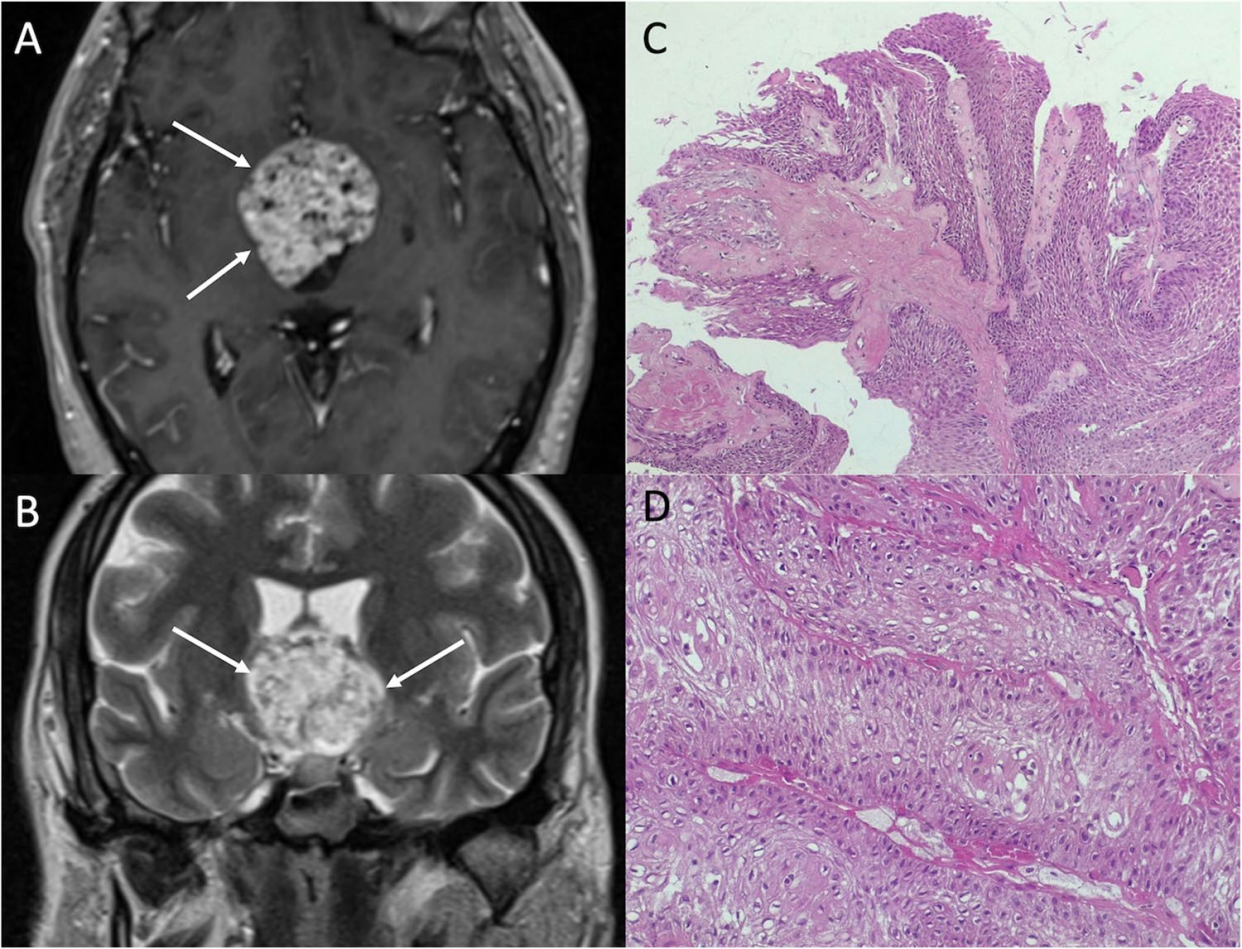
Papillary craniopharyngioma. Axial contrast-enhanced T1- (**A**) and coronal T2-weighted (**B**) MR images show a suprasellar expansile lesion compressing the third ventricle floor featuring vivid enhancement and small cystic components (arrows). Microscopic examination shows a squamous non keratinizing epithelium lining a fibro-vascular core. Squamous whorls are present in the upper pole of the microscopic field (hematoxylin-eosin staining, original magnification 100×) (**C**). At higher magnification, the squamous nature of epithelial tumor cells can be seen in more detail (hematoxylin-eosin staining, original magnification 400×) (**D**)

PCPs occur almost exclusively in adults at 45–60 years of age, with symptoms similar to those described for ACPs and mainly related to their mass effect such as headache, visual disturbance and hydrocephalus, as well as endocrine deficit [47]. On MRI, they typically appear as suprasellar or intraventricular solid, noncalcified mass with smooth margins. Cysts are less common than ACPs, and if present they appear as smaller, less complex and with a clear fluid content, not linked to any spontaneous T1-weighted hyperintensity. After gadolinium administration, PCP usually shows a relatively homogeneous enhancement (Fig. 8). Main PCP differential diagnoses include germ cell tumors, which are usually characterized by a pronounced diffusion restriction and a vertical infundibular extension, and pituitary metastases.

Pathological examination shows the presence of a well differentiated squamous, non-keratinizing epithelium, lining a fibro-vascular core [52].

For both ACP and PCP, surgical resection with open or endoscopic endonasal approaches represents the primary treatment [53]. A cyst drainage catheter is occasionally inserted to decompress cystic ACPs. In case of incomplete removal, stereotactic radiotherapy or radiosurgery are commonly used to prevent residual lesion growth. Pituitary hormonal deficits secondary to the tumor itself, surgery or radiotherapy can be managed with hormonal replacement therapy. Intracavitary chemotherapy and immunotherapy represent further therapeutic options in case of ACP recurrence, while targeted therapy has provided new perspectives for treatment of PCP harboring BRAF p.V600E mutation [54]

### Germ cell tumors

**Fig. 9:**
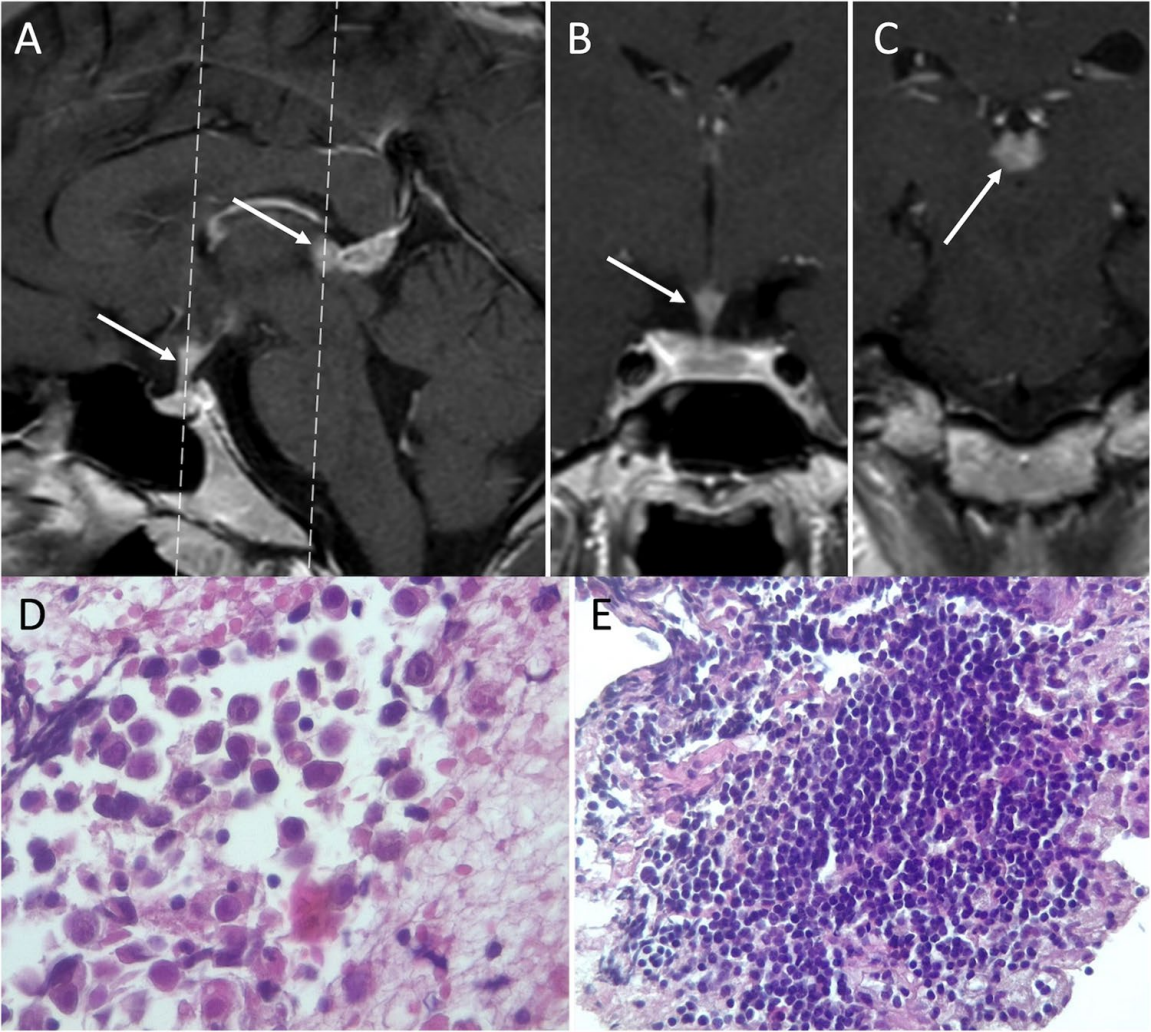
Germinoma. Sagittal contrast-enhanced T1-weighted image (**A**) shows thickened enhancing pituitary infundibulum and habenular commissure (arrows). Coronal images corresponding to the dotted lines in A confirm this finding (**B**, **C**). On histology, a neoplasm composed of large-sized, polygonal cells arranged in lobules or “sheets” is evident, with vesicular nuclei featuring conspicuous nucleoli (hematoxylin-eosin staining, original magnification 400×) (**D**). A chronic lymphoplasmacytic infiltrate is also visible, especially in perivascular areas (hematoxylin-eosin staining, original magnification 630×) (**E**). Tumor cells are immunoreactive for PLAP, c-KIT, and negative per CK-pan and S100 protein (not shown)

**Fig. 10:**
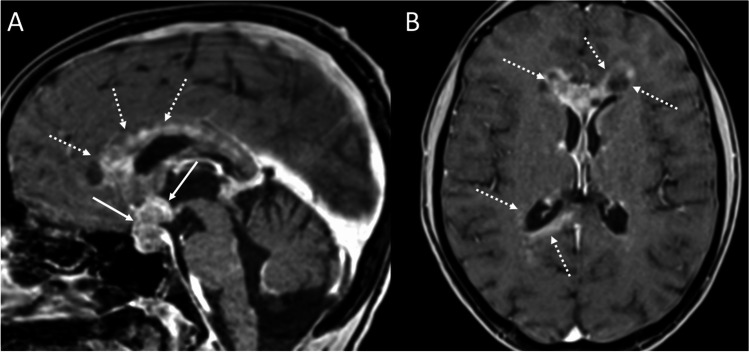
Germinoma with diffuse perivascular spread. Sagittal (**A**) and axial (**B**) contrast-enhanced T1-weighted images reveal an intra- and suprasellar mass along with diffuse corpus callosum and periventricular tumoral spread

Intracranial germ cell tumors (IGCTs) are a heterogeneous group of rare pediatric neoplasms, with peak presentation at 10–14 years of age and a peculiar predilection for the midline, with pineal, and suprasellar region involvement [55]. The first clinical manifestation is often diabetes insipidus, usually occurring before lesions become visible on MRI [56]. Disease progression then result in the development of visual loss, hypothalamic-pituitary dysfunction (with decreased growth and precocious puberty), obstructive hydrocephalus, and in late cases metastatic dissemination via CSF [57]. IGCTs can be divided in two groups, containing respectively germinomas and non-germinomatous germ cell tumors (NGGCTs). The latter group encompasses a large number of tumors, including embryonal carcinoma, yolk sac tumor, choriocarcinoma, teratoma, and mixed IGCTs, with some of these that might produce tumor markers (such as b-HCG or AFP) that can be detected in both serum and CSF and can be helpful in indicating the histological subtypes. Germinomas (WHO grade 2), that represent two-thirds of IGCTs [58], appear on MRI as a well-demarcated infundibular mass (although bifocal -infundibular and pineal- presentation is also often present) with a iso-hyperintense signal on T1-weighted and a slightly low signal intensity on T2-weighted sequences due to the high nuclear to cytoplasmic ratio. Moreover, the loss of the normal pituitary bright spot on T1-weighted sequence is a typical feature of sellar germinomas [59]. Variably sized intratumoral cysts, especially in larger lesions, and calcifications may also be present, with gadolinium enhancement that is strong and usually homogeneous (Fig. 9). Given of their hypercellularity and high nuclear to cytoplasmic ratio, germinomas show restricted diffusion on DWI that results in lower ADC values than in other sellar neoplasms. Finally, they may show extensive peritumoral T2/FLAIR hyperintensity mimicking granulomatous inflammatory disorders like neurosarcoidosis and histiocytosis, and may spread by direct infiltration of the surrounding brain parenchyma or remotely via CSF (Fig. 10).

On the other hand, NGGCTs are usually larger than germinomas, often causing obstructive hydrocephalus, and show the presence of spontaneous T1-hyperintense foci with a moderate lesion enhancement. On CT, germinomas are uniformly hyperdense, while NGGCTs appear more inhomogeneous, with particular reference to mature teratomas that appear as mixed density lesions with large cysts and areas of calcification.

In the presence of a pineal mass, the differentiation between IGCTs and pineoblastoma is not easy, because of their similar density and signal intensity, as well as the shared presence of cystic components. Nevertheless, the bifocal presentation is highly suggestive of IGCTs.

On histology, germinoma appears as a neoplasm composed of large-size, polygonal cells arranged in lobules or “sheets,” with vesicular nuclei having conspicuous nucleoli. A chronic lymphoplasmacytic infiltrate can be visible, especially in perivascular areas, with tumor cells are immunoreactive for PLAP, c-KIT, and negative per CK-pan and S100 protein [60].

With reference to treatment, germinomas are significantly radiosensitive and responsive to chemotherapy treatments [61]. Mature teratomas are usually treated with surgery, while the other NGGCTs are usually managed with a combination of surgery, chemotherapy, and radiotherapy depending on the tumor type. Molecular targeted therapy with inhibitors of KIT/RAS activation and the AKT1/mTOR pathway potentially represent promising therapeutic strategies [62]. Prognosis is usually good for germinomas and mature teratomas, while choriocarcinomas and embryonal carcinomas are often associated with a poorer outcome.

### Primary posterior pituitary tumors

Primary posterior pituitary tumors (PPTs), in the revised definition of the 2021 WHO classification, are rare slow-growing neoplasm (WHO grade 1) of middle-aged adults, that arise from different morphological variants (dark and light cells, oncocytic, granular, and ependymal types) of the pituicytes of infundibular stalk and neurohypophysis and representing a spectrum of the same entity. They typically show immunopositivity for the thyroid transcription factor-1 (TTF-1) and include pituicytoma (PC), spindle cell oncocytoma (SCO), granular cell tumor (GCT), and sellar ependymoma (SEP) [33].

**Fig. 11:**
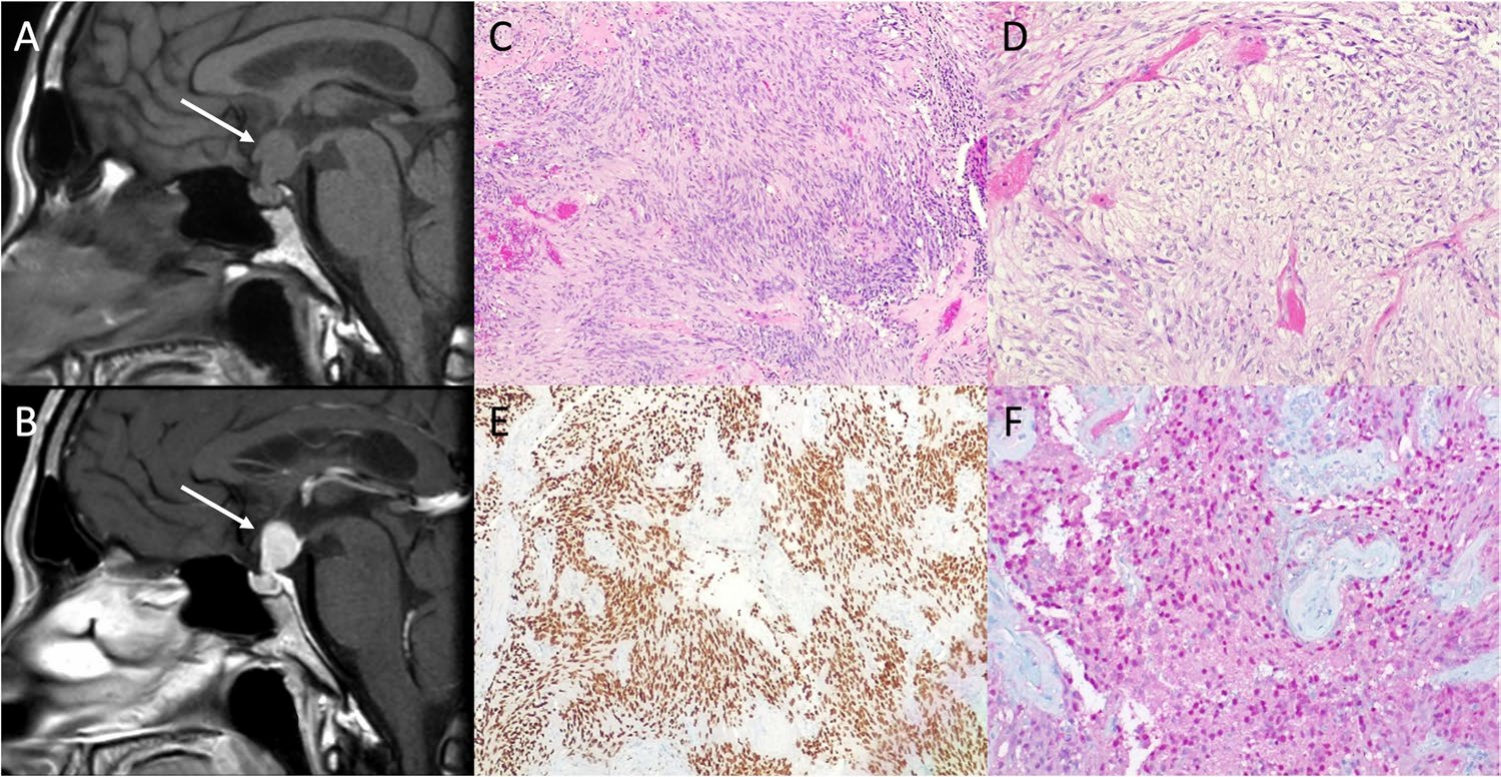
Pituicytoma. Sagittal T1- (**A**) and contrast-enhanced T1-weighted (**B**) MR images show a suprasellar enhancing solid lesion (arrows) compressing the third ventricle chiasmatic and infundibular recesses. The neoplasm is predominantly composed of bipolar spindle cells arranged in solid sheets and short fascicles, with elongated nuclei and abundant cytoplasm, often organized in fibrillary, anucleate areas (hematoxylin-eosin staining, original magnification 100×) (**C**). In some areas, more epithelioid cells with clear-cell cytoplasm are evident (hematoxylin-eosin staining, original magnification 200×) (**D**). Tumor cells are positive to immunostaining for TTF1 (immunoperoxidase staining, original magnification 100×) (**E**). Moreover, S100 immunostaining shows strong and diffuse positivity (immunoperoxidase staining, original magnification 200×) (**F**). The S100 immunoreactivity can lead to a misdiagnosis of schwannoma, which is conversely TTF1-

**Fig. 12:**
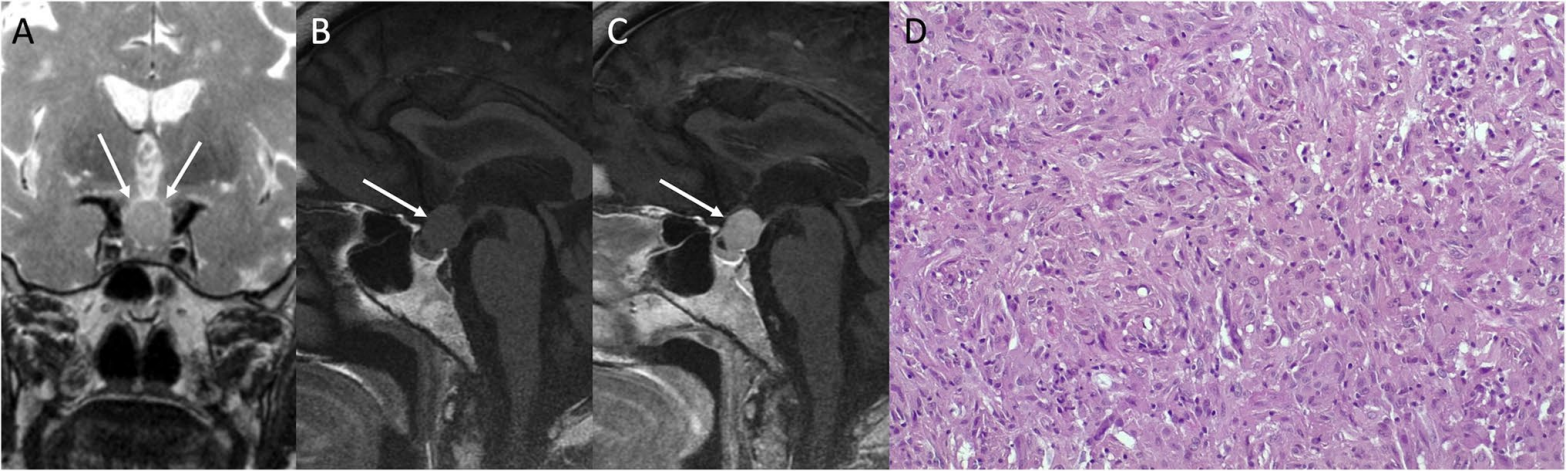
Spindle cell oncocytoma. Coronal T2- (**A**), sagittal T1- (**B**), and contrast-enhanced T1-weighted (**C**) MR sequences show an intra- and suprasellar solid lesion compressing the third ventricle floor (arrows). Histological images reveal a tumor with solid architecture, composed of interlacing fascicles of spindle and epithelioid cells, with mild pleomorphic nuclei, prominent nucleoli, and eosinophilic to oncocytic cytoplasm (hematoxylin-eosin staining, original magnification 400×) (**D**). On immunohistochemistry (not shown) tumor cells are S100+, TTF1+, vimentin+, EMA−, GFAP−, as well as negative for all pituitary hormones

The main clinical manifestations resemble that of NFPAs and include visual defects, hypopituitarism, and headache. Diabetes insipidus is uncommon. Acute presentation with pituitary apoplexy is extremely rare [63]. CT usually reveal an enhancing solid homogenous mass in the sellar and/or suprasellar region often paired with a slightly enlargement of the sella. On MRI, PPTs generally appear as solid well circumscribed infundibular masses isointense to hypointense on T1- and isointense to hyperintense on T2-weighted sequences, with avid and homogenous enhancement (Figs. 11 and 12). An early and rapid enhancement on dynamic contrast-enhancement MRI is typical and due to a well-developed capillary network [64]. PC can be located either in the sellar or suprasellar region and is the only PPT which may present as a purely intrasellar mass. SCOs usually appear as sellar masses with suprasellar extension, typically contain multiple T2-hypointense foci and linear flow void areas. Anterior displacement of the pituitary gland and cavernous sinus extension are common features. GCTs present as solid intra-suprasellar lesions, intimately associated with the infundibulum, and mainly iso-hypointense on T2-weighted images. However, purely cystic GCTs have been reported. The “star-like crack sign,” consisting in converging linear enhancing structures within the lesion, has been suggested as a specific feature of these neoplasms [65].

PC is predominantly composed of bipolar spindle cells arranged in solid sheets and short fascicles, with elongated nuclei and abundant cytoplasm, often organized in fibrillary, anucleate areas. In some areas can be present more epithelioid cells with clear-cell cytoplasm. The strong and diffuse S100 immunoreactivity can lead to a misdiagnosis of schwannoma (TTF1−). SCO has a solid architecture, composed of interlacing fascicles of fusated and epithelioid cells, with mild pleomorphic nuclei, prominent nucleoli, and eosinophilic to oncocytic cytoplasm. On immunohistochemistry tumor cells are S100+, TTF1+, vimentin+, EMA−, GFAP−, and negative for all pituitary hormones.

PPTs are often misdiagnosed before surgery and the most common radiological preoperative diagnosis is a PA. Differential diagnoses also include CP, germinoma, lymphocytic hypophysitis, histiocytosis, neurosarcoidosis, and metastases.

Complete resection is important to prevent tumor recurrence but is often challenging due to the fibrous and vascular nature of these tumors, and most patients require revision surgery and/or adjuvant radiotherapy. The optimal management strategy includes therapeutic presurgical angiography with tumor feeders’ embolization to reduce intraoperative bleeding, and subsequent gross total resection via craniotomy or endoscopic transsphenoidal surgery. Anti-VEGFR therapy and somatostatin analogs could be a potential option for the treatment of PPTs [66].

### Hypothalamic hamartomas

Hypothalamic hamartomas (HHs) represent nonneoplastic congenital gray matter heterotopia arising from the tuber cinereum, a hypothalamic region located between the mammillary bodies and the infundibulum. It is related to neuronal migration abnormalities which probably occur between gestational days 33 and 41 [67]. The size of the lesion varies from a few millimeters to huge mixed solid-cystic lesions measuring several centimeters in diameter. Two types of HHs have been described: parahypothalamic or pedunculated and intrahypothalamic or sessile. Presenting symptoms include precocious puberty, due to tumoral neurosecreting properties, developmental delay and gelastic epilepsy, which is characterized by seizures of laughter and represents the most frequent isolate manifestations of hypothalamic hamartomas [68].

**Fig. 13:**
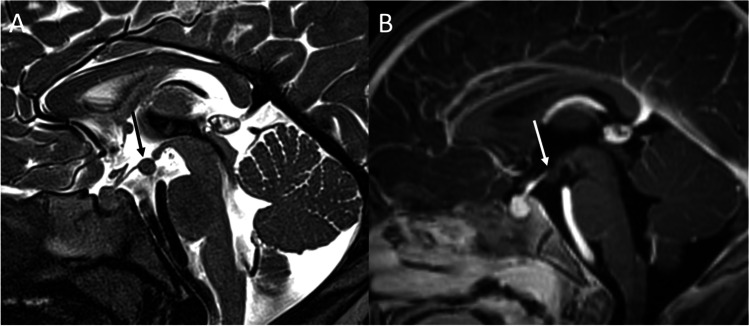
Hypothalamic hamartoma. Sagittal T2- (**A**) and contrast-enhanced T1-weighted (**B**) images show a non-enhancing sessile lesion of the tuber cinereum

MR imaging accurately depict this entity, showing a sessile or pedunculated mass mainly isointense to the gray matter centered in the tuber cinereum region, without contrast enhancement (Fig. 13).

The major differential diagnosis is represented by hypothalamic-chiasmatic glioma, which tends to be T1-hypointense and T2-hyperintense, often showing contrast enhancement.

HHs management may require surgery in drug-resistant seizures. Central precocious puberty is successfully treated with pharmacological therapy as luteinizing hormone-releasing hormone agonists.

### Rathke cleft cyst

Rathke cleft cyst (RCC) is a benign lesion located within or above the pituitary gland, arising from remnants of the fetal Rathke pouch [69]. These lesions vary in size from a few millimeters up to 40 mm, with the majority measuring between 10 and 20 mm in diameter. Most RCCs are asymptomatic, and the majority discovered incidentally. Clinical manifestations include headache, pituitary dysfunction, and visual disturbances, due to compression [70].

**Fig. 14:**
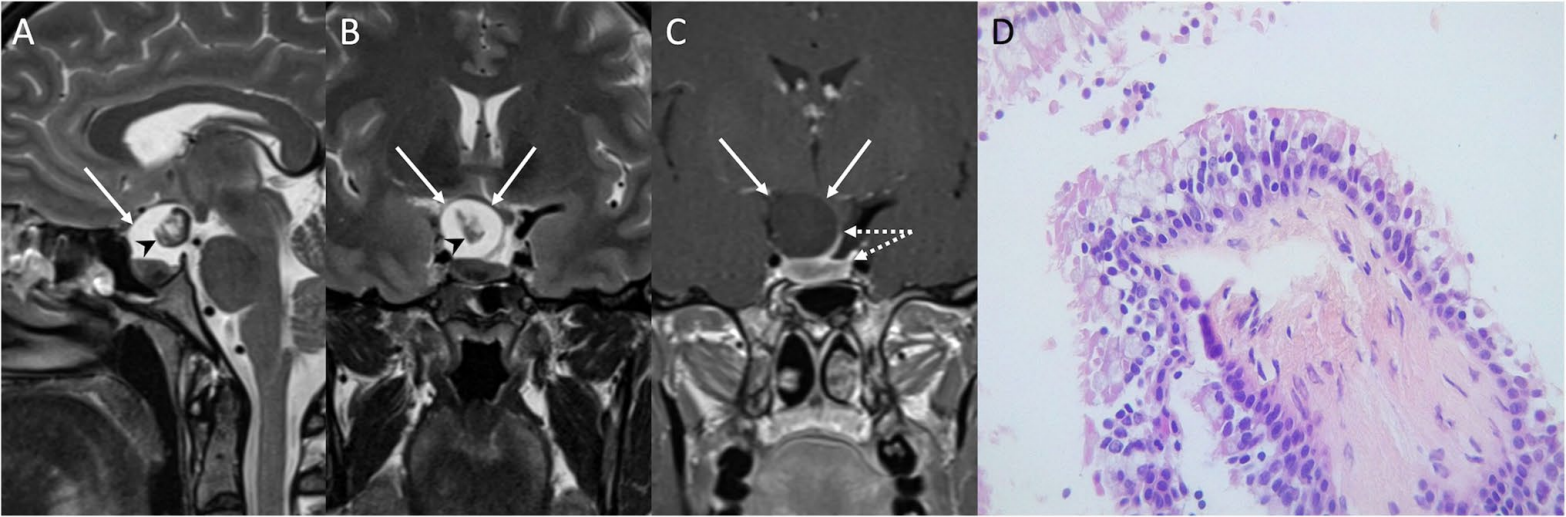
Rathke cleft cyst. Sagittal T2- (**A**), coronal T2- (**B**), and contrast-enhanced T1-weighted (**C**) MR images show a suprasellar cystic lesion (arrows) with a non-enhancing intracystic nodule (arrowheads). The lesion is dissociable from the pituitary gland and its stalk (dotted arrows). The latter is displaced to the left. Histological examination reveals small fragments of fibrovascular stroma, partially lined by a pseudostratified columnar epithelium resembling that of respiratory tract. Intermingled goblet cells are also evident (hematoxylin-eosin staining, original magnification 400×) (**D**)

CT is useful for diagnosis, showing a well-demarcated round or ovoid intrasellar-suprasellar mass located at the midline between the anterior and posterior pituitary lobes. RCCs usually appear homogeneously hypodense (75% of the cases), less frequently mixed hypo-isodense (20%) and hyperdense (10%), without calcifications. They may show ring-like or capsular enhancement. MR signal depends on the cyst content. On T1-weighted images, RCCs may present both hypo- or hyperintense; on T2-weighted sequences, about 70% appear hyperintense while 30% are iso-to-hypointense [71]. An almost pathognomonic feature for RCC is the presence of an intracystic, nonenhancing nodule, showing low intensity on T2- and high intensity on T1-weighted imaging compared to cyst fluid (Fig. 14) [72]. After contrast administration, an enhancing rim of compressed pituitary surrounding the cyst may give rise to the “claw sign.”

On histology, RCC shows small fragments of fibrovascular stroma, partially lined from pseudostratified columnar epithelium, resembling that of respiratory tract, with intermingled goblet cells.

Main differential diagnoses include CPs and PAs. CPs often show enhancing solid components and calcifications, not present in RCCs. On the other hand, intracystic nodule, midline location within the gland, no fluid-fluid level, and no septation may help differentiate RCCs from PAs.

RCCs are usually stable “leave me alone” lesions with a good prognosis. Surgical treatment may be required in case of symptomatic lesions or diagnostic doubts.

### Arachnoid cyst

Arachnoid cysts (ACs) represent the most common off-midline extra-axial cysts and are usually located within the subarachnoid spaces, including the suprasellar cistern.

Also known as meningeal cysts, they are CSF filled lesions lined by a layer of flattened arachnoid cells. ACs are sometimes in communication with subarachnoid cisterns.

Most ACs are primary and due to meningeal development anomalies (embryonic endomeninges fail to merge and remain separated). On the other hand, inflammation, tumor, trauma, or hemorrhage may give rise to secondary ACs [73].

Although ACs are generally asymptomatic, some patients may experience a wide variety of symptoms related to the location and size of the lesion. Nevertheless, the most frequent clinical presentation is headache. Subdural hematoma may represent a rare complication of ACs after minor trauma [74].

**Fig. 15:**
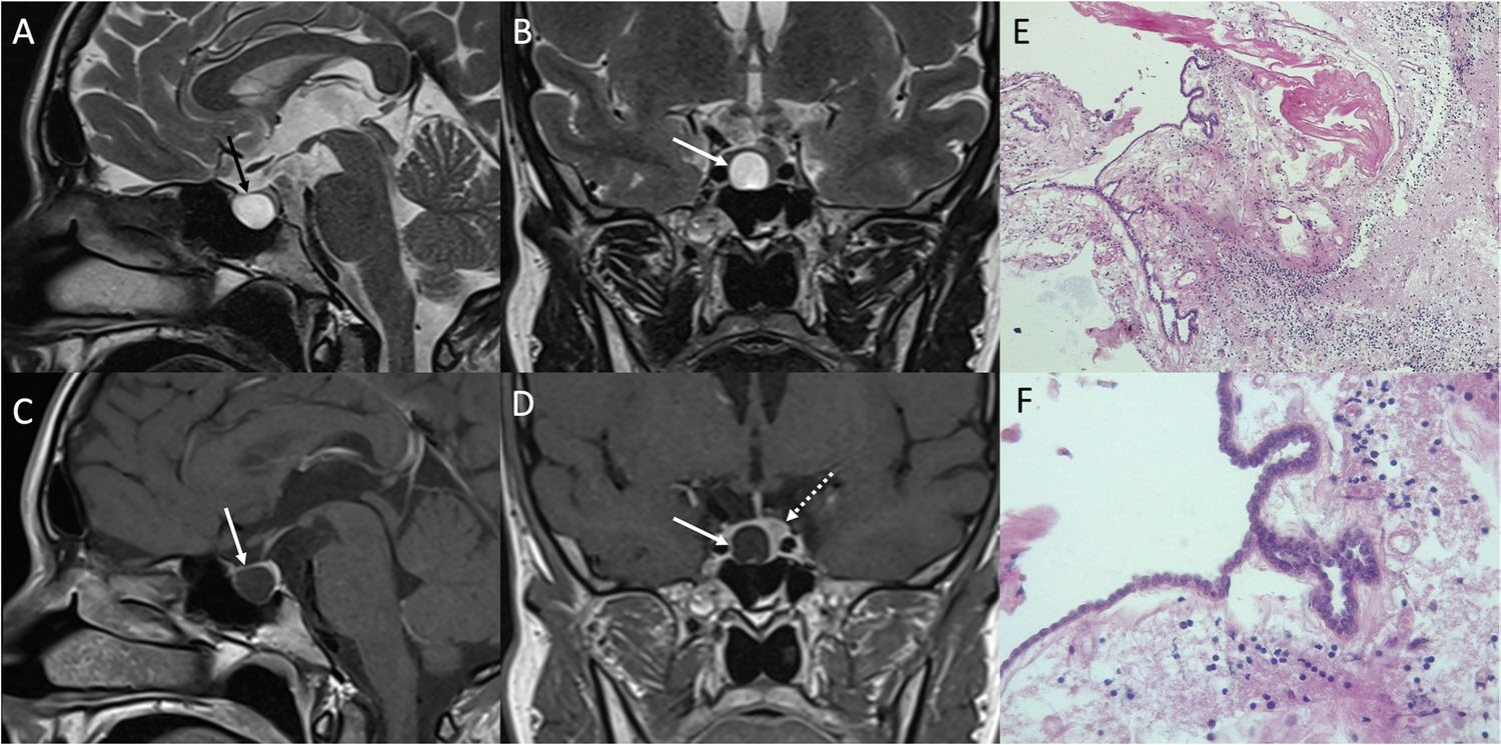
Arachnoid cyst. Sagittal (**A**) and coronal (**B**) T2-, sagittal (**C**), and coronal (**D**) contrast-enhanced T1-weighted MR images demonstrate the presence of an intrasellar cystic lesion (arrows), displacing the pituitary gland to the left (dotted arrow). Histological examination shows a delicate fibrous tissue forming the cyst walls (arachnoid laminar connective tissue), lined by a thin meningothelium. The stroma appears edematous with lymphocytic infiltrate (hematoxylin-eosin staining, original magnification 100×) (**E**). Higher magnification highlighting epithelial cells (hematoxylin-eosin staining, original magnification 400×) (**F**)

On CT, uncomplicated ACs show CSF density and do not enhance. Intrathecal instillation of contrast agent (CT cisternography) may be helpful in documenting communication with the subarachnoid space. ACs also follow CSF signal on all MRI sequences (Fig. 15). They do not present diffusion restriction nor contrast enhancement. Suprasellar ACs may compress chiasm, pituitary stalk, and/or the third ventricle floor.

Histological examination show a delicate fibrous tissue forming cyst wall (arachnoid laminar connective tissue) lined by an attenuated meningothelium. Stroma is edematous with lymphocytic infiltrate.

The main differential diagnosis is the epidermoid cyst which however shows higher signal than CSF on FLAIR sequence and diffusion restriction.

Asymptomatic ACs do not require treatment (“leave me alone” lesions). When treatment is required, surgical options include fenestration, endoscopic resection, marsupialization, or cysto-peritoneal shunting with a programmable valve.

### Hypophysitis

**Fig. 16:**
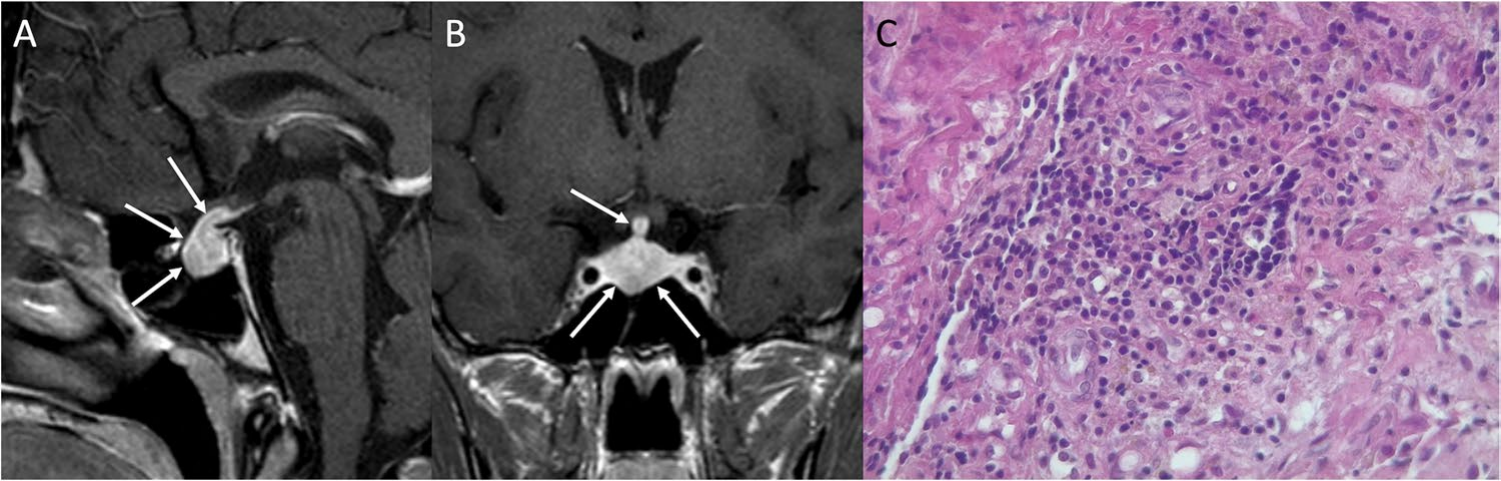
Lymphocytic hypophysitis. Sagittal (**A**) and coronal (**B**) contrast-enhanced T1-weighted MR images reveal a symmetric enlargement of the pituitary gland extending cranially along the stalk and compressing the infundibular recess (arrows). The histological examination shows a fragment of fibrotic tissue with lymphoplasmacytic infiltrate. The background is hemorrhagic with pigmented macrophages (hematoxylin-eosin staining, original magnification 400×) (**C**)

Hypophysitis is a rare inflammatory disease of the pituitary gland and its stalk that can be classified based on the anatomical extension (adenohypophysitis, infundibuloneurohypophysitis, panhypophysitis), its etiology (primary or secondary, when associated with sellar lesions or systemic inflammatory diseases), and histopathology [75]. Lymphocytic, or autoimmune, hypophysitis (LHy) is the most common form of primary hypophysitis and occurs more frequently in women during late pregnancy or the postpartum period [76]. Granulomatous hypophysitis (GHy) and xanthomatous hypophysitis (XHy) have a middle-aged female predilection, without relation with pregnancy. GHy may occur as primary disease or can be secondary to systemic granulomatous diseases (sarcoidosis, granulomatosis with polyangiitis, tuberculosis, histiocytosis). According to some authors, GHy may be a late histopathological manifestation of LHy. IgG4-related, or plasmacytic, hypophysitis (IgG4Hy) is usually part of a systemic disease involving multiple organs and tends to develop in males in the seventh decade of life. On the other hand, isolated forms of IgG4Hy seem to be more common in females. Furthermore, recently identified entities of autoimmunity-related paraneoplastic endocrine syndromes have been reported: anti-PIT1 hypophysitis, isolated ACTH deficiency, and immune checkpoint inhibitor-related hypophysitis [77]. Headache and visual disturbances are the most common presenting symptoms due to mass effect. Other clinical features include anterior pituitary hormone deficiencies with a typical sequential order (ACTH, followed by TSH, LH/FSH, GH) [78], central diabetes insipidus, and hyperprolactinemia. Hypophysitis may also have an acute onset, with pituitary apoplexy, circulatory collapse, and adrenal crisis. MRI findings suggestive of hypophysitis include a diffuse and symmetrical gland enlargement (triangular- or dumb-bell-shaped) with thickened stalk, loss of posterior pituitary bright spot in T1-weighted images, and intense homogenous enhancement, with a symmetrical suprasellar extension (Fig. 16) [79, 80]. Furthermore, a very useful feature in the diagnosis of hypophysitis is the presence of parasellar T2 dark sign. It has been shown that dark-signal-intensity areas on T2 images around the pituitary gland and in the cavernous sinus are very suggestive of hypophysitis and useful for distinguishing PAs from LHy [81]. GHy may show a more heterogeneous enhancement. XHy often presents as a cystic sellar mass with peripheral contrast-enhancement. Adjacent dural linear thickening and enhancement, as well as sphenoid sinus mucosal swelling are typical of LHy. In later stages, pituitary gland fibrosis and atrophy may result in “empty sella” [82].

Histological examination shows fragment of fibrotic tissue with lymphoplasmacytic infiltrate and a hemorrhagic background with pigmented macrophages.

Differential diagnoses include NFPAs, CPs, metastases, RCC, IGCTs, lymphoma and, in the post-partum period, Sheehan’s syndrome. The management of hypophysitis consists of high-dose glucocorticoid administration and hormone replacement therapy [83]. Moreover, during the acute phase of hypophysitis, immunosuppressive therapy should be promptly started to reduce the compressive effects of the enlarged pituitary gland which may lead to an irreversible hypopituitarism [76]. Surgery is generally reserved for refractory hypophysitis with persistent mass effect not responding to glucocorticoid or in case of progressive symptom worsening.

### Pituitary abscess

Pituitary abscess represents a rare but life-threatening condition, with immunosuppression, surgery, and infections that are known predisposing factors [84]. The clinical presentation includes fever, meningism, and symptoms due to mass effect, such as headache, visual change, and hypopituitarism [85]. On MRI, a cystic or partially cystic pituitary mass with the typical features of abscess is usually found (namely, high T2-weighted signal, diffusion restriction with low ADC values, and peripheral/rim enhancement [86]). Signs of adjacent anatomic structures involvement, such as a stalk thickening or a meningeal enhancement are also often present [86]. The most common differential diagnoses are hemorrhagic or necrotic macroadenomas, RCCs, ACPs, pituitary apoplexy, and LHy, with their treatment that is surgical (drainage of the abscess through a trans-sphenoidal approach, followed by antibiotic treatment [85]).

### Sellar region lymphoma

**Fig. 17:**
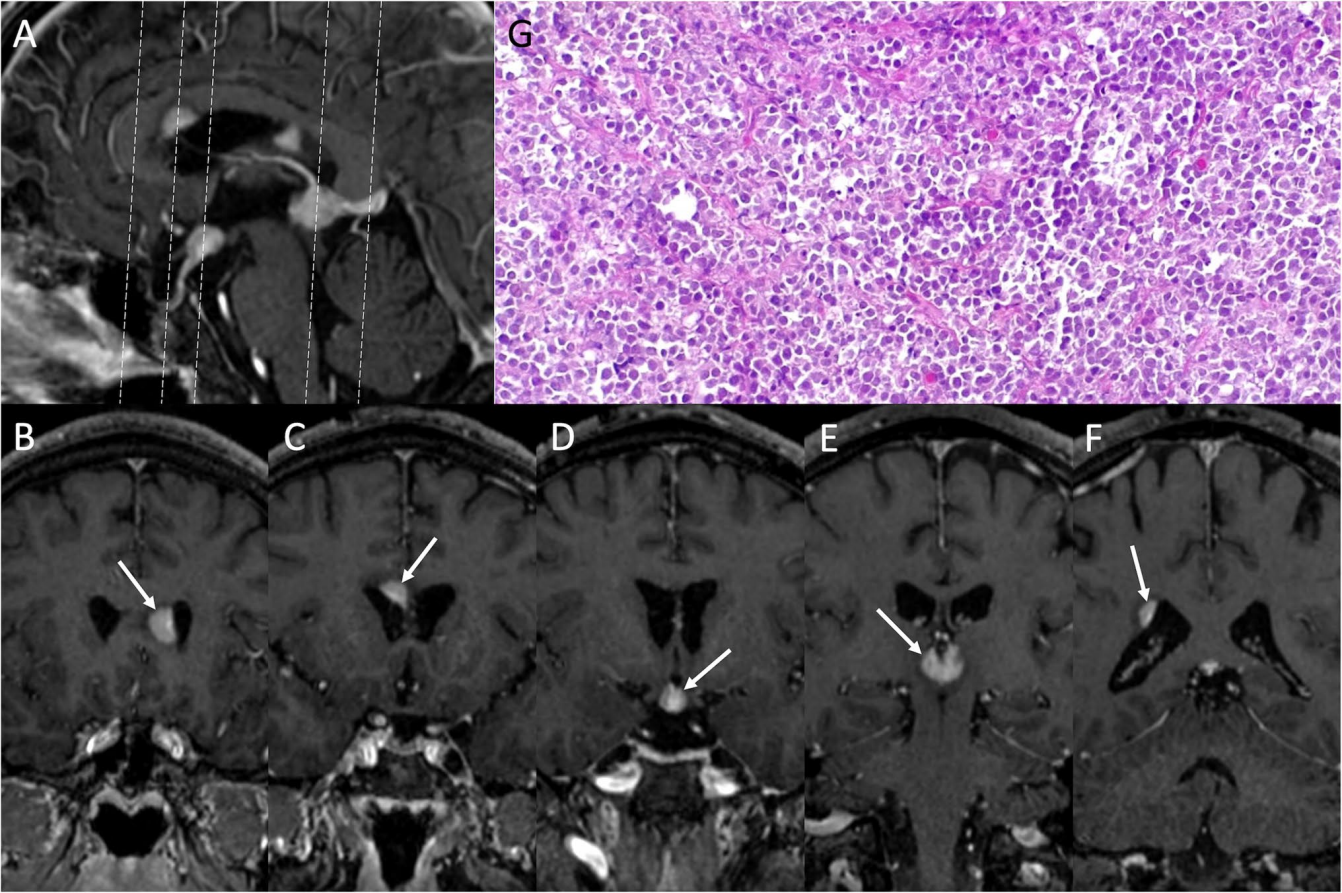
Lymphoma. Sagittal (**A**) and coronal (**B**–**F**) contrast-enhanced T1-weighted MR images show multiple periventricular enhancing nodules (arrows) of which one localized at the level of the infundibulum. On hematoxylin-eosin staining, a tumor composed of medium to large sized cells, arranged in a discohesive pattern, is evident (hematoxylin-eosin staining, original magnification 200×). The immunohistochemical profile (not shown) is consistent with a high-grade B cell lymphoma

Lymphoma of the sellar region is a rare entity that may origin from the infundibulum, pituitary gland, or the hypothalamus, with a diffusely infiltrative growth and a propensity for perineural spread. Diffuse large B cell lymphoma represents the most common type (90%), and usually occurs in immunodepressed patients with a mean age of 30–40 years. However, in the last years, its incidence has been increasing in immunocompetent patients, with a peak presentation in the sixth decade of age and male predilection [87]. The typical clinical features of sellar lymphomas include systemic symptoms of lymphoma (fever, night sweat, and weight loss), symptoms related to the mass effect and to cranial nerve impairment, such as headache, diplopia, visual field defects. The optic nerve is the most frequently involved, followed by the third and the sixth cranial nerve. The sequential order of anterior pituitary hormone deficiencies follows diminished gonadotropin secretion and then GH, TSH, and ACTH deficiency. Hyperprolactinemia, due to stalk compression, and diabetes insipidus are often present. Lymphomas are also the most common neoplasm causing fever of unknown origin [88]. MR imaging usually reveals a purely intrasellar or sellar with a suprasellar extension mass, iso- to hypointense on T1-weighted images and T2-weighted images. There is often erosion of the sellar floor with invasion of cavernous sinus and/or sphenoidal sinus with a permeative pattern. The absence of hyperintensity on T2-weighted images is due to both the high cellularity of these neoplasm, that results also in a typical diffusion restriction on DWI, and the high nucleus-to-cytoplasm ratio, which also leads to hyperdensity on CT [89]. Multiple lesions can present in both immunocompetent and, more frequently, immunodepressed patients (Fig. 17). Furthermore, contrast enhancement is intense and homogeneous in immunocompetent patients, while in immunodepressed patients is more likely to be inhomogeneous or ring-like, a feature of the high degree of necrosis [90].

On hematoxylin-eosin staining, these tumors are composed of medium to large-sized cells, arranged in a discohesive pattern, and show an immunohistochemical profile consistent with a high-grade B cell lymphoma.

Differential diagnoses include infections, metastases, and PAs. The role of surgery is generally restricted to stereotactic biopsy. The management of lymphomas is based on multimodal treatment, which includes high-dose methotrexate, other chemotherapeutic agents and radiotherapy [91]. Unfortunately, disease recurrence is commonly observed.

### Meningioma

**Fig. 18:**
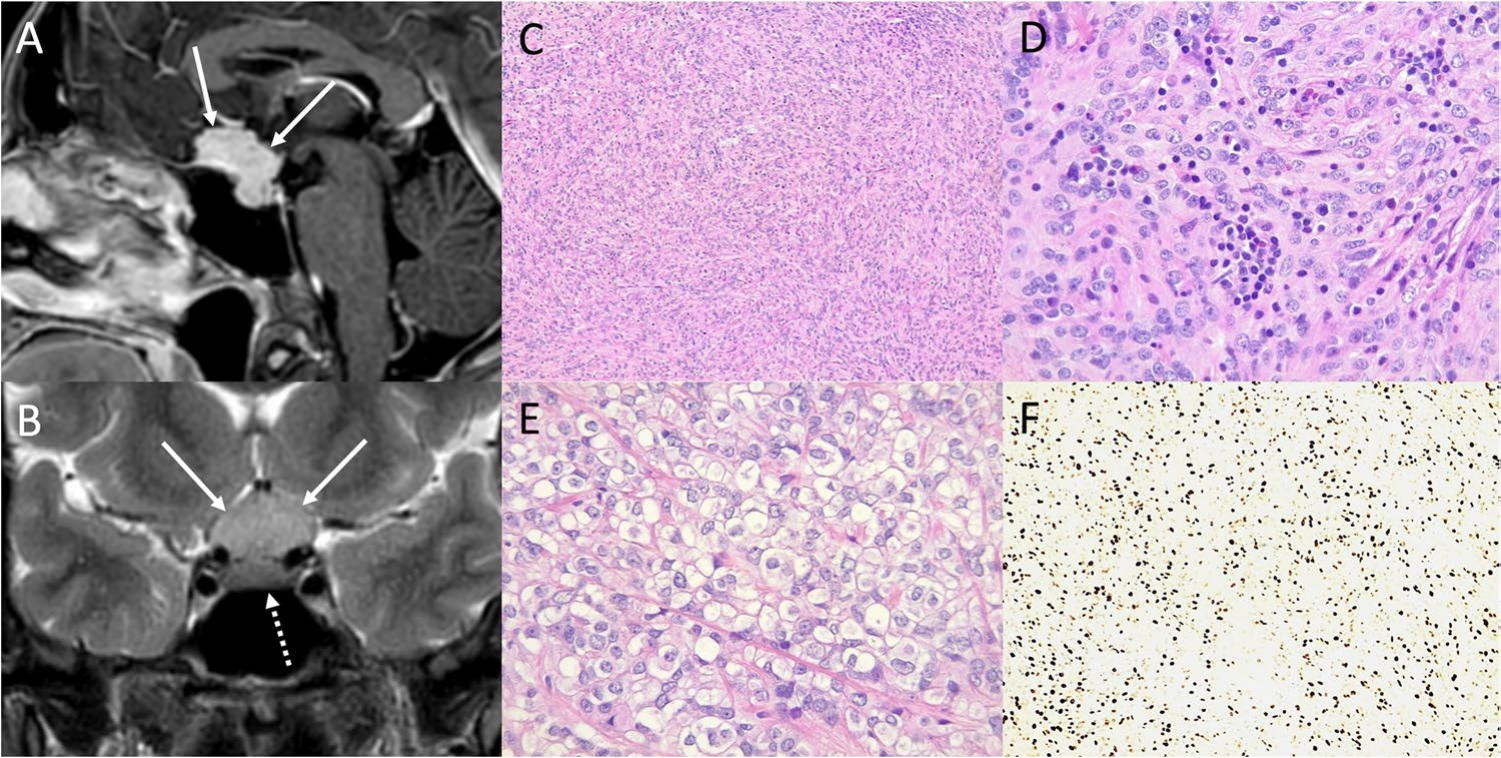
Tuberculum sellae meningioma. Sagittal contrast-enhanced T1- (**A**) and coronal T2-weighted MR images demonstrate a dural-based expansile lesion of the tuberculum sellae (arrows) extending into the sellar and suprasellar compartments, dissociable from the pituitary gland (dotted arrow). Histological examination shows a neoplasm with loss of lobular architecture (“sheeting”) and hypercellularity (hematoxylin-eosin staining, original magnification 100×) (**C**). In some field, cells with high nuclear/cytoplasmatic ratio (“small cell change”) are visible (hematoxylin-eosin staining, original magnification 200×) (**D**), along with nodular areas composed of clear cells (hematoxylin-eosin staining, original magnification 200×) (**E**). Immunolabeling with Ki67 shows a proliferative index near to 30% (immunoperoxidase staining, original magnification 100×) (**F**)

Meningioma is the second most common tumor of the sellar region in adults, with an incidence peak between the sixth and the seventh decade of life and female predominance. Meningiomas of this region may arise from the tuberculum sellae, clinoid processes, or cavernous sinus walls. Clinical presentation is related to compression of the surrounding structures, such as visual impairment, endocrine dysfunction, or cranial nerve deficits. On MRI, meningioma appears as a well circumscribed, round or lobulated, extra-axial dura-based mass. It enhances homogenously and brightly [92]. T2-hyperintensity is often associated with a soft consistency, while hypointense tumors tend to be more fibrous and firmer. Reactive thickening of the adjacent dura (“dural tail”) extending away from the tumor tissue is often present but is not pathognomonic of meningioma [93]. CT is more sensitive to detect calcifications and hyperostosis of the adjacent skull base. Tuberculum sellae meningiomas (TSMs) characteristically lie in a suprasellar sub-chiasmal midline position displacing the optic chiasm posteriorly and superiorly and the optic nerves laterally when invading optic canals, causing progressive visual loss (Fig. 18). Clinoidal meningiomas (CLM), frequently centered on the anterior clinoid processes, rapidly invade the optic canal compressing the nerve with unilateral progressive visual loss and headache. Large CLMs may secondarily extend to the superior orbital fissure and cavernous sinus, leading to additional cranial neuropathies such as diplopia and facial hypoesthesia [94]. Cavernous sinus meningiomas (CSM) originate from the lateral dural wall of cavernous sinus and slowly extent within the sinus, leading to stenosis of the intracavernous segment of the internal carotid artery and cranial nerve disjunctions. Meningiomas tend to narrow the lumen more than other tumors encasing blood vessels. Adjacent hyperostosis or expansion into the sphenoid sinus are often present [95].

On histological examination, atypical meningiomas show loss of lobular architecture (“sheeting”) and hypercellularity. In some field, cells with high nuclear/cytoplasmatic ratio (“small cell change”) can be visible, along with nodular areas composed of clear cells. Immunolabeling with Ki67 shows a proliferative index near to 30%. Tumor cells are EMA+, and nuclear staining for progesterone receptor is nearly 30%.

Differential diagnoses of sellar/parasellar meningiomas include metastasis, lymphoma, PA, and cavernous sinus venous malformation. Surgery is the therapy of choice in symptomatic or growing lesions. However, the proximity of these tumors to the optic nerve, cavernous sinus, and internal carotid artery often precludes safe, complete resection. Adjuvant radiotherapy and stereotactic radiosurgery can be used in selected cases of macroscopic residual after surgical resection. Cavernous sinus invasion is one of the main factors limiting the extent of resection.

### Cavernous venous malformations

**Fig. 19:**
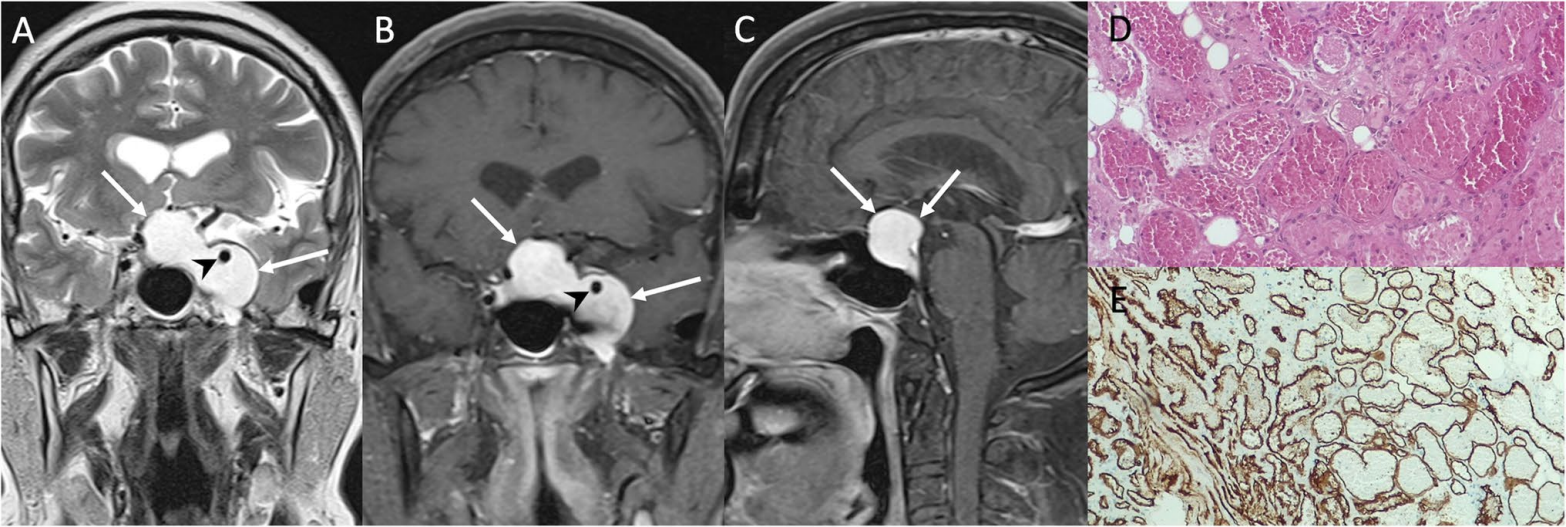
Cavernous sinus venous malformation. Coronal T2- (**A**), coronal (**B**) and sagittal (**C**) contrast-enhanced T1-weighted MR sequences reveal an expansile lesion involving the left cavernous sinus (arrows), encasing the ipsilateral internal carotid artery (arrowheads), and extending into the sellar and suprasellar compartments, displacing the pituitary gland. The lesion is highly hyperintense on T2-weighted images and displays homogeneous enhancement. On microscopy, different sized, ectasic, vascular channels, often back-to-back arranged and densely-packed, are evident. Vessels are lined by flattened, benign-appearing endothelium. (hematoxylin-eosin staining, original magnification 200×) (**D**). The immunostaining for CD31 is distinctly positive (immunoperoxidase staining, original magnification 100×) (**E**)

**Fig. 20:**
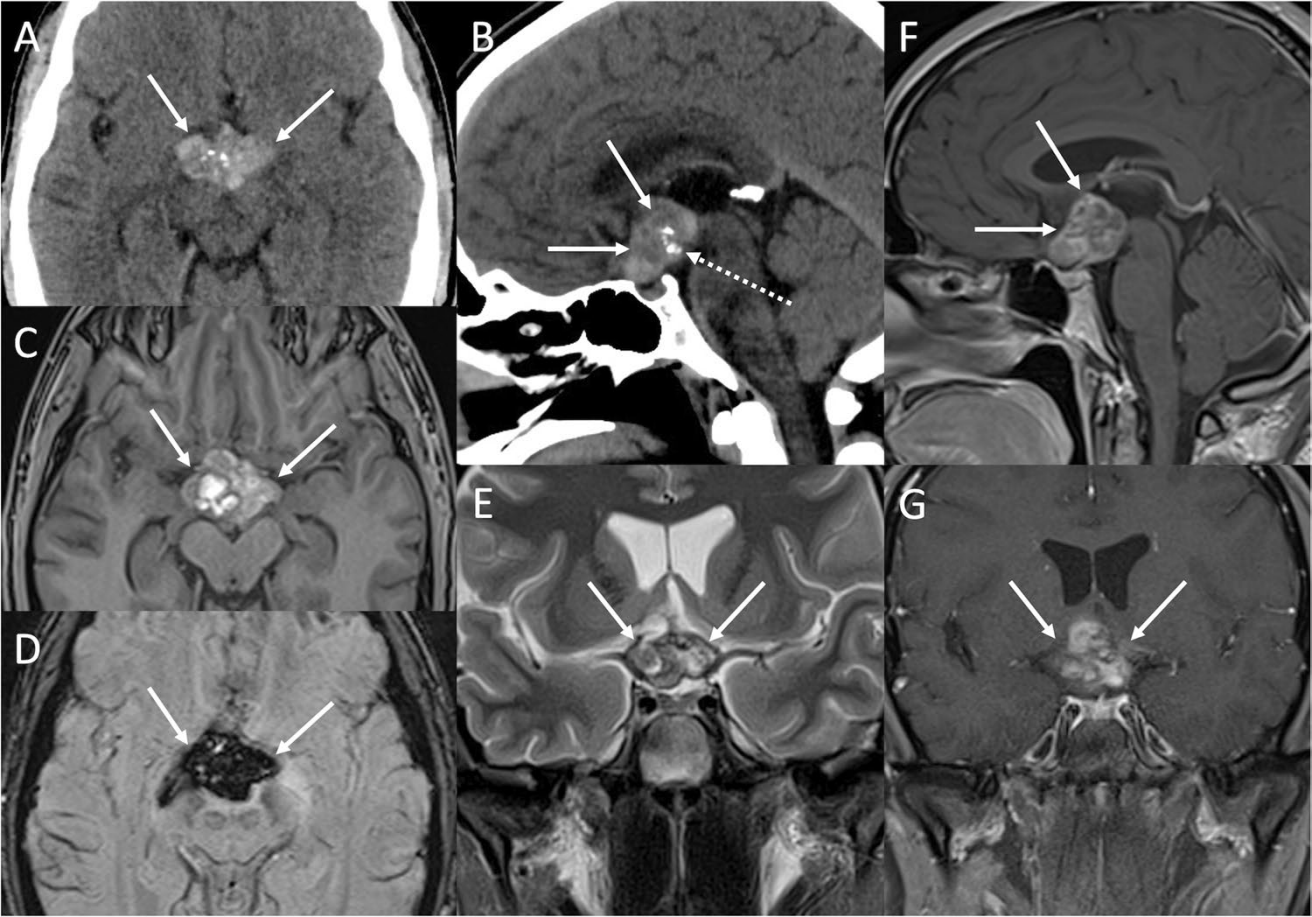
Chiasmatic cavernous venous malformation. Axial (**A**) and sagittal (**B**) CT reformats show a suprasellar multilobulated mass (arrows), slightly hyperdense with small dystrophic intralesional calcifications (dotted arrow). The lesion presents high signal on T1- (**C**), low signal on SWI (**D**), and appears inhomogeneous on T2-weighted (**E**) MR images. Mild capsular enhancement is evident on sagittal (**F**) and coronal (**G**) contrast-enhanced T1-weighted sequences

Cavernous hemangiomas of cavernous sinus are not true neoplasms and are better described as venous malformations (VMs) according to the International Society for the Study of Vascular Anomalies (ISSVA) 2018 classification. VM is a rare congenital vascular anomaly containing endothelial-lined sinusoidal spaces filled with slow-flowing or stagnant blood. Although all VMs are present at birth, they may not manifest until late childhood or young adulthood, occurring more commonly in middle-aged women. Clinical manifestations include headache, visual impairment, and cranial nerve palsies. VMs of the cavernous sinus tend to grow as asymmetrical dumbbell-shaped mass extending into the sellar region or middle fossa with three patterns of growth: endophytic lateral growth, with extension into the middle cranial fossa, endophytic medial growth, with medial extension into the sella or anterior extension into the superior orbital fissure, and exophytic growth from the sinus wall [96]. On MRI, VMs classically appear as well-defined polylobulated masses, highly T2-hyperintense (Fig. 19). Dynamic contrast-enhanced T1-weighted images show characteristic delayed slow centripetal “fill-in” of contrast agent. Low T2-signal nuclei within VMs may be caused by the presence of hemosiderin, calcifications, phleboliths, or vascular channels (Fig. 20) [97].

On microscopy, VMs show different sized, ectasic, vascular channels, often back-to-back arranged, densely packed. Vessels are lined by flattened, benign-appearing endothelium. The immunostaining for CD31 is typical.

Differential diagnoses include PA, meningioma, and schwannoma. Total removal of these highly vascular lesions is often very difficult due to the risk of severe intraoperative bleeding and the presence of critical neurovascular structures within the cavernous sinus. Stereotactic radiosurgery has been shown to be a successful primary or adjuvant strategy to decreasing lesion volume.

### Pituitary metastasis

**Fig. 21:**
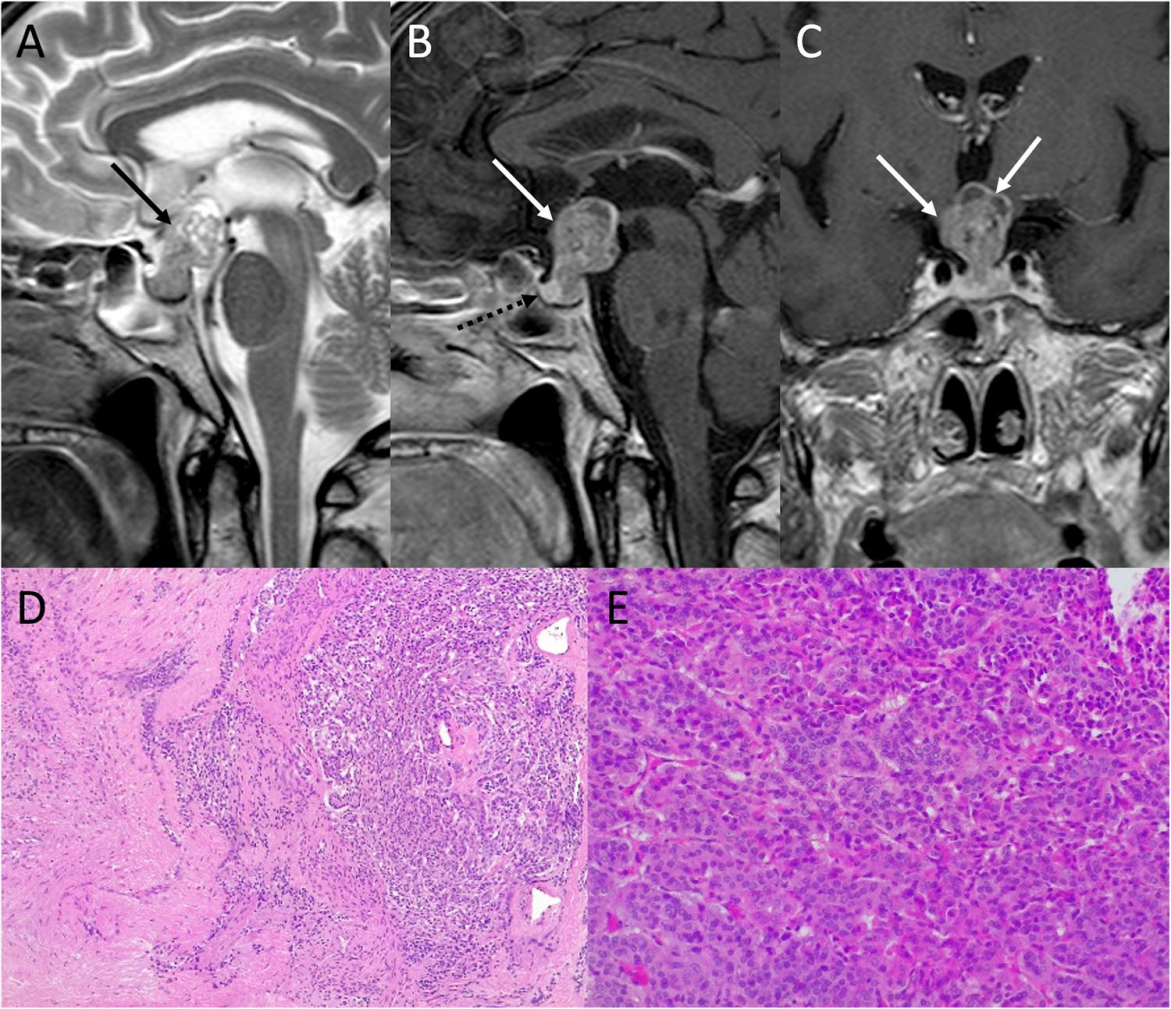
Pituitary metastasis. Sagittal T2- (**A**), sagittal (**B**), and coronal (**C**) contrast-enhanced T1-weighted images reveal an intra- and suprasellar mass (arrows) presenting small cystic components, more evident at its cranial aspect. Pituitary gland is recognizable and displaced anteriorly (dotted arrow). On hematoxylin-eosin staining, at low magnification, a tumor with a follicular growth pattern surrounded by fibrous stroma is depicted (hematoxylin-eosin staining, original magnification 100×) (**D**). Follicular structures are scanty, tumor architecture being predominantly solid/nested. Tumor cells present round-to-oval nuclei with fine chromatin and small, centrally located nucleoli (hematoxylin-eosin staining, original magnification 200×) (**E**). On immunohistochemistry (not shown) the tumor is TTF1+, thyreoglobulin^+^, PAX8^+^, CK19^−^, CD56^−^, CDX2^−^, a pattern consistent with a thyroid carcinoma metastasis

Pituitary metastases are rare (about 1% of all surgically treated pituitary lesions) [98]. Lung and breast are the most common primary sites of origin, with a predilection for the posterior pituitary, given its direct connection with systemic circulation [99]. The onset of diabetes insipidus in an oncologic patient is highly suggestive of pituitary metastasis [100]. Hypopituitarism, headache, and visual disturbances are less specific symptoms. On MRI, pituitary metastases usually manifest as T1 hypo- to isointense and T2 hyperintense, contrast enhancing intra/suprasellar mass. Other typical features are the absence of posterior pituitary T1-bright spot, or a stalk thickening (Fig. 21). Cavernous sinus invasion and bone erosion are less common.

The histologic findings vary according to the histotype of the primary tumor.

Differential diagnoses include LHy, lymphoma, Langerhans cell histiocytosis, and neurosarcoidosis. Treatment and prognosis depend on the primary tumor. Surgical decompression and radiotherapy can be considered in symptomatic patients.

### Optic pathway glioma

**Fig. 22:**
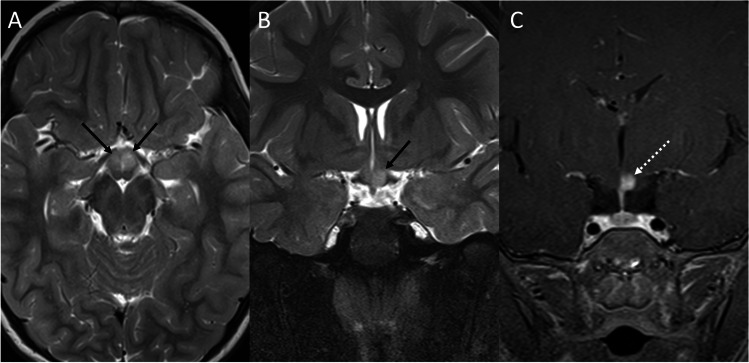
Hypothalamic glioma. Axial (**A**) and coronal (**B**) T2-, and coronal contrast-enhanced T1-weighted (**C**) sequences show a T2-hyperintense lesion (arrows) presenting a nucleus of enhancement (dotted arrow)

Optic pathway gliomas (OPGs) are rare low-grade gliomas (WHO grade 1) that arise from the optic pathway (optic nerve, optic chiasma, optic tract, lateral geniculate body) or hypothalamus, most frequently in children with a mean age of 4–5 years. The main histological subtypes are pilocytic astrocytoma and pilomyxoid astrocytoma. Pilocytic astrocytoma is often bilateral, arising from the optic nerve, and is frequently observed in patients affected by neurofibromatosis type 1 (NF1), while other subtypes are mostly sporadic and located posteriorly. Pilomyxoid astrocytoma may have an aggressive behavior, with a tendency to metastasize via CSF. OPGs in children with NF1 usually have a better prognosis. Onset symptoms are insidious and include visual impairment, pituitary/hypothalamic dysfunction, or hydrocephalus. For this reason, OPGs are often diagnosed late, noticing a visual disturbance progression or exophthalmos for tumors arising from the orbital segment of optic nerve, or nystagmus for OPGs involving the optic chiasm or hypothalamus [101]. On MRI, OPGs tend to manifest as T2-hyperintense suprasellar masses, with moderately heterogeneous enhancement (Fig. 22). Large tumors may present cystic components [102].

On histopathology, pilocytic astrocytomas show round to spindled nuclei and dendrite-like cytoplasmic processes, often paired with Rosenthal fibers.

The main differential diagnosis is optic nerve meningioma, that is frequently calcific. OPGs in patients with NF1 sometimes show a spontaneous regression [103]. Management strategies include surgery, chemotherapy, and radiotherapy. Surgery is rarely curative and is reserved to tumors causing severe symptoms related to their mass-effect such as hydrocephalus.

### Anterior communicating artery aneurysm

**Fig. 23:**
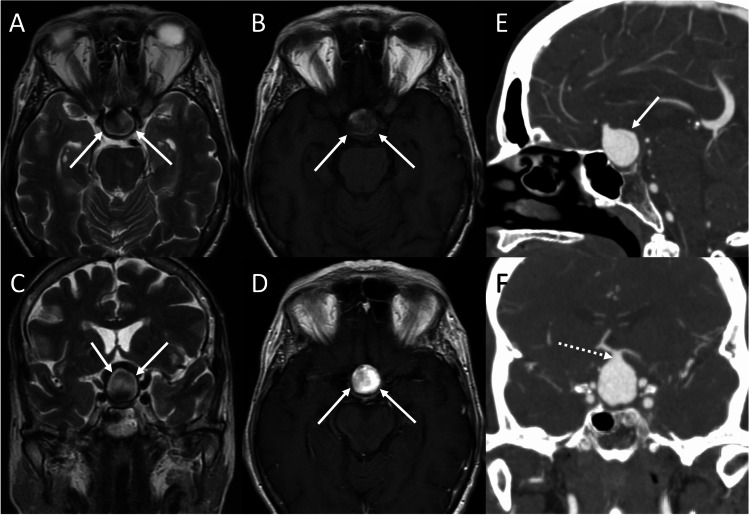
Anterior communicating artery aneurysm. Axial T2- (**A**) and T1- (**B**), coronal T2- (**C**), and axial contrast-enhanced T1-weighted (**D**) MR images display an intra- and suprasellar round expansile lesion (arrows) featuring inhomogeneous T1- and T2-signal due to flow artifact and presenting homogeneous enhancement. Sagittal (**E**) and coronal (**F**) CT angiography reformats confirm the presence of a saccular aneurysm whose neck is located at the level of the anterior communicating artery (dotted arrow)

Aneurysms arising from anterior communicating arteries (ACoA) can mimic a suprasellar or parasellar mass. Saccular aneurysms are the most common type. Peak presentation is between 40 and 60 years of age, with a female predominance. Typical manifestations are related to mass effect and include visual impairment due to optic chiasm compression. Giant aneurysms (> 2.5 cm) may extend into the sellar cavity mimicking pituitary tumors and resulting in hypopituitarism, due to pituitary/pituitary stalk compression [104]. ACoA aneurysms present as heterogeneous, round suprasellar masses, mildly hyperdense on non-contrast CT scan. Rim or mural calcification may be present. Giant aneurysms can cause bone scalloping. On MRI, aneurysms have dark signal intensity on T2-weighted images due to flow void. Pulsation artifacts can also be seen in phase-encoding direction. In case of partially or completely thrombosed aneurysms, they show heterogenous signal, often with clot hyperintensity on T1-weighted images. Furthermore, aneurysms can present heterogeneous signal intensity secondary to slow or turbulent flow, saturation effects, and phase dispersion (Fig. 23). “Blooming” artifact on SWI is usually observed. Microsurgical or endovascular treatments are selected in relation to the aneurysm characteristics (shape and size), anatomy of the adjacent blood vessels and perforating arteries.

## Conclusions

Differential diagnosis of neoplasms and tumor-like lesions of the sellar region may not always be easy because of the plethora of entities possibly involving this anatomical region, some of which presenting with similar findings on MRI. The radiologist must be aware of the presence of this wide range of differential diagnoses for proper diagnostic framing. In addition, knowledge of the latest updates on their classification and related pathologic findings contributes to a better understanding of these entities and to appropriately interface with other specialists within the multidisciplinary team assessment.

## Abbreviations

*ACP*: Adamantinomatous craniopharyngioma
*CP*: Craniopharyngioma
*CSF*: Cerebrospinal fluid
*CT*: Computed tomography
*DWI*: Diffusion-weighted imaging
*GCT*: Granular cell tumor
*IGCT*: Germ cell tumor
*MRI*: Magnetic resonance imaging
*NF1*: Neurofibromatosis type 1
*NFPA*: Non-functioning pituitary adenoma
*NGGCT*: Non-germinomatous germ cell tumor
*OPG*: Optic pathway glioma
*PA*: Pituitary adenoma
*PC*: Pituicytoma
*PCP*: Papillary craniopharyngioma
*PitNET*: Pituitary neuroendocrine tumor
*PPT*: Primary posterior pituitary tumor
*SCO*: Spindle cell oncocytoma
*SEP*: Sellar ependymoma
*SWI*: Susceptibility-weighted imaging
*TTF-1*: Thyroid transcription factor-1
*WHO*: World Health Organization
